# Molecular phylogenetics and systematics of two enteric helminth parasites (*Baylisascaris laevis* and *Diandrya vancouverensis*) in the Vancouver Island marmot (*Marmota vancouverensis*)

**DOI:** 10.1016/j.ijppaw.2022.11.006

**Published:** 2022-11-17

**Authors:** McIntyre A. Barrera, Jasmine K. Janes, Jamieson C. Gorrell

**Affiliations:** Biology Department, Vancouver Island University, Nanaimo, BC, V9R 5S5, Canada

**Keywords:** Phylogeography, Coevolution, Island biogeography, Parasite conservation, Cryptic biodiversity

## Abstract

Island biogeography can promote rapid diversification and speciation via geographic isolation and novel selection pressures. These same factors can threaten the persistence of island endemics by limiting gene flow and suitable habitat. Host-parasite interactions on islands introduce another dimension of complexity as both species must simultaneously adapt to exogenous and endogenous factors. One example of host-parasite island biogeography is the critically endangered Vancouver Island (VI) marmot (*Marmota vancouverensis*) which is endemic to VI, Canada, and hosts two enteric helminth parasites: *Baylisascaris laevis*, an ascarid nematode common in tribe Marmotini, and *Diandrya vancouverensis*, an anoplocephalid cestode endemic to the VI marmot. Here, we aligned novel sequences from *B. laevis* (six genes) and *D. vancouverensis* (two genes) with congeneric sequences from GenBank. Phylogenies reconstructed using Bayesian and maximum parsimony approaches consistently placed *B. laevis* in a morphoclade, and *D. vancouverensis* in a monophyletic clade sister to *D. composita*. Mean pairwise sequence divergence between *D. vancouverensis* and *D. composita* (9.06 ± 1.94%) surpassed commonly accepted thresholds for species delimitation, whereas divergence between VI and mainland populations of *B. laevis* (1.12 ± 0.78%) was comparable to (or sometimes greater than) pairwise divergence values between other *Baylisascaris* species. Disparity in the genetic divergence of each parasite may reflect differences in their life cycle, host specificity, virulence, and the chronological extent of their isolation. Detailed descriptions of the population genetic structure and effects of both parasites on their shared host are crucial next steps in understanding the history of *B. laevis* and *D. vancouverensis* on VI and informing conservation efforts for the VI marmot and its enteric helminth parasites.

## Highlights

• First nDNA and mtDNA sequences for *Baylisascaris laevis* and *Diandrya vancouverensis.*
• *B. laevis* was sister to *B. procyonis* (raccoon).
• *B. laevis* from Vancouver Island was divergent from the mainland.
• *Diandrya vancouverensis* was a unique species, endemic to *Marmota vancouverensis.*
• Parasites of endangered hosts are also at risk and represent cryptic biodiversity.

## Graphical abstract

**Figure:**
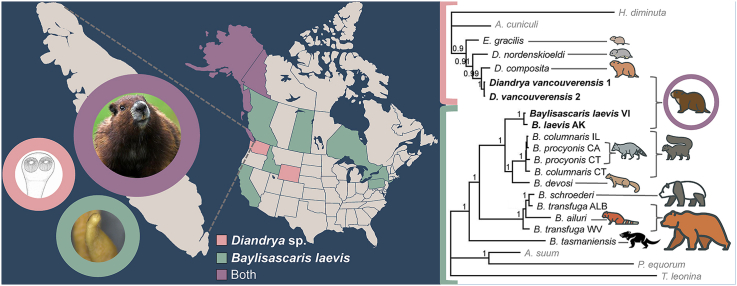

## 1 Introduction

Island biogeography is a major driver of speciation, with geographically isolated ecosystems providing hotspots of endemism. The dynamic factors that promote speciation and persistence of insular endemics include neutral models of evolution as well as adaptation to habitat-specific selection pressures (Whitehead and Jones, 1969; Eldridge et al., 2018). Isolated populations can diverge over generations by chance fluctuation in allele frequencies when gene flow is interrupted. The process of divergence is accelerated when small founder populations colonize islands, importing a small subset of the total genetic variation from their source population, and these events are an important driver of speciation on islands (Matzke, 2014). Host-parasite associations can introduce unique selection pressures, wherein factors such as frequency-dependent selection and co-adaptation contribute additional dimensions of evolutionary complexity (Gandon, 2002; Goater et al., 2014). Anthropogenic habitat destruction, pollution, and climatic influences threaten these fragile dynamics at an accelerating rate, increasing the need for formal description of biodiversity to inform conservation efforts (Leclerc et al., 2020). Vancouver Island (VI) constitutes one potential hotspot of cryptic biodiversity as several unique populations have been described (Walser et al., 2005; Chavez et al., 2014; Hessels et al., 2021), with at least two diverging to the extent of speciation (Swarth, 1911; Taylor et al., 2012).

The distribution of parasites within host taxa follows an oscillatory pattern, with co-speciation interspersed by periodic host-switching (Hoberg and Brooks, 2008). The extent to which parasites mirror host evolution is influenced by life cycle characteristics, including host specificity and mode of dispersal. In some cases, parasite phylogeography mirrors the host, as they colonize novel habitats together (Carlson et al., 2021), but parasites can undergo host-independent speciation events when their associations with hosts are unstable (Štefka et al., 2011). At the microevolutionary level, the frequency of host-switching events is negatively related to the extent of co-adaptation between a parasite and its hosts (Gandon, 2002).

Parasites impose unique selection pressures by influencing host fitness, and the nature of their influence is determined by qualities of the parasite itself (Goater et al., 2014). Differences in the specific reproductive and dispersal characteristics of parasite species, along with the biogeographic and climatic context of their association may influence their degree of co-adaptation and co-speciation with hosts (Gandon, 2002). Enteric helminths, including monoecious cestodes and dioecious nematodes, are endoparasites with diverse life cycles, which can exert a range of effects on intermediate and definitive hosts (Castro, 1996).

The Vancouver Island marmot (*Marmota vancouverensis* Swarth, 1911; VIM) is endemic to VI and is listed as Critically Endangered by the IUCN and Endangered by the Committee on the Status of Endangered Wildlife in Canada (COSEWIC, 2019). It is the youngest, and the only insular *Marmota* species, thought to have diverged rapidly from its sister species, the hoary marmot (*M. caligata*), following post-glacial colonization of VI 12–13 thousand years ago (Kya) (Nagorsen and Cardini, 2009; Kerhoulas et al., 2015). The VIM is host to two enteric helminth parasites: an ascarid nematode *Baylisascaris laevis* Leidy, 1856 and an anoplocephalid cestode *Diandrya vancouverensis* Mace and Shepard (1981). Genetic sequence data have been unavailable for either species, hence both have been excluded from molecular phylogenetic analyses within their respective taxa. The effect of parasite burden on VIM health has yet to be examined in detail, and the extent to which these two helminth species have diverged from their mainland counterparts has never been investigated.

**Fig. 1:**
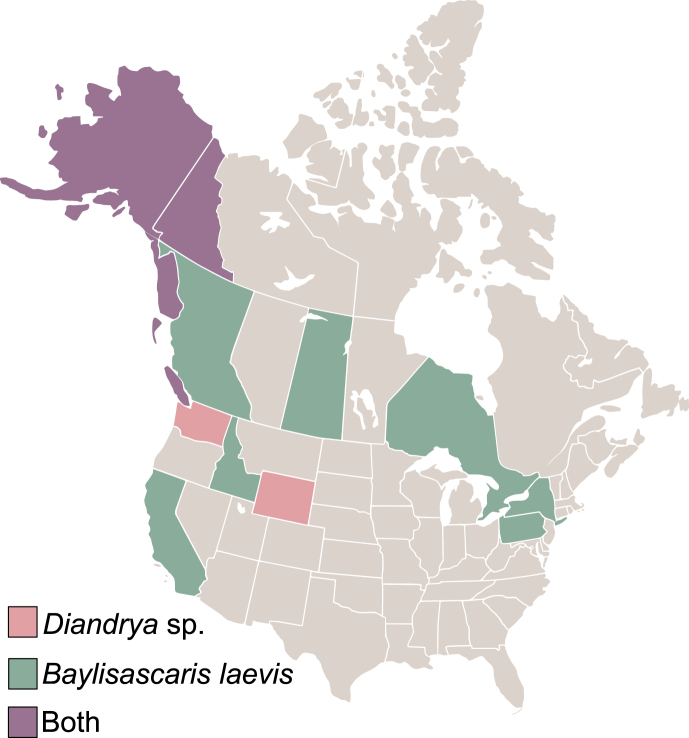
Known geographic distributions of *Baylisascaris laevis* and *Diandrya composita* in North America.

*Baylisascaris laevis* is known to parasitize members of the Marmotini tribe, including *Marmota*, *Urocitellus*, and *Otospermophilus*, and has been recorded across Canada (ON, SK, BC) and the United States (AK, NY, PA, and CA) (Fig. 1; Berry, 1985; Sapp et al., 2017). It is the only member of the genus *Baylisascaris* with rodent definitive hosts, as well as the only species without an intermediate host —transmission occurs directly between definitive hosts via eggs ingested during grooming or feeding (Berry, 1985; Sapp et al., 2017) — although *B. procyonis* can also be transmitted vertically by the same means (Kazacos, 2001). Visceral pathology in naturally and experimentally infected hosts has been demonstrated as larvae migrate within host tissues (Babero, 1960). Camp et al. (2018) provides the most recent, and comprehensive, molecular systematic analysis of *Baylisascaris,* including seven of the 11 recognized species in the genus, but excluding *B. laevis.* The phylogenetic reconstruction shows two clades — the first containing species from gulonine mustelid, skunk, and raccoon hosts; the second encompassing species that parasitize marsupials, bears, and red panda (*Ailurus fulgens*) (Camp et al., 2018). Historically, Sprent (1968) placed *B. laevis* with *B. columnaris* (from skunks) and *B. procyonis* (from raccoons) based on morphology.

*Diandrya vancouverensis* differs from its sister species, *D. composita* Darrah (1930), based on morphology. No additional infections of *D. vancouverensis* have been reported in the literature since its description, with subsequent cestode infections in *M. vancouverensis* (perhaps erroneously) attributed to *D. composita* (Raverty and Black, 2001)*. Diandrya composita,* on the other hand, is known to occur in four of the other five *Marmota* species in North America (all except the woodchuck *M. monax*), and has been reported in Alaska, Yukon, Washington, and Wyoming (Fig. 1; Rausch and Rausch, 1971). The complete life cycle of *D. composita* has not been described; however, it is hermaphroditic (Rausch, 1980), and is presumed to rely on free-living mites as intermediate hosts, as is common for cestodes of the family Anoplocephalidae (Rausch and Rausch, 1971; Denegri, 1993). The phylogenetic relationships among *D. composita* and other anoplocephalid cestodes have been resolved using genetic sequence data (Wickström et al., 2005), whereas the phylogenetic placement of *D. vancouverensis* has yet to be assessed beyond morphological comparisons.

Here, we compared newly generated nuclear and mitochondrial sequences from *B. laevis* and *D. vancouverensis*, collected from VI and mainland North America, to existing sequences from related species. We aimed to: 1) infer the phylogenetic placement of *B. laevis* within *Baylisascaris*; 2) assess the extent of divergence between *B. laevis* from VI and the mainland; and 3) resolve species-level relationships among *D. vancouverensis* and *D. composita*.

## 2 Materials and Methods

### 2.1 Specimen acquisition and DNA extraction

The Marmot Recovery Foundation provided adult specimens of *Baylisascaris* sp. and *Diandrya* sp. from VIM fecal samples. Mainland tissue samples of adult *Baylisascaris* sp. were collected during necropsy of *Urocitellus columbianus* in Idaho (Cook et al., 2017) and *Marmota monax* in Alaska (Table 1). Three Idaho samples were contributed by the Division of Parasites, Museum of Southern Biology, University of New Mexico (MSBP 24571, 24572, and 24594). The sole Alaskan sample was contributed by the University of Alaska's Museum of the North (UAM 130585). We were unable to locate samples from *M. caligata*. All specimens were preserved in ethanol and *Baylisascaris* specimens were presumed to be *B. laevis* based on their host species. DNA extraction followed the manufacturer's protocol using a Qiagen DNeasy® Blood & Tissue Kit (Qiagen, Toronto, Ontario, Canada). For *Diandrya*, sections of proglottid were incubated at 56 °C in 180 μl Buffer ATL and 20 μl Proteinase K for 3 h. For *Baylisascaris*, sections of tail or midsection were incubated overnight, for enhanced digestion of cuticle-bound tissue. DNA extracts were resuspended in 100 or 200 μl of Buffer AE and stored at −20 °C.

**Table 1:** GenBank accession numbers for *Baylisascaris* and outgroup sequences included in the analysis. The length of each alignment is listed in nucleotide base pairs. Specimens sequenced in this study were contributed by the Marmot Recovery Foundation (MRF), the University of Alaska Museum of the North (UAM), and the Division of Parasites, Museum of Southwestern Biology (MSBP), University of New Mexico. Specimens from the MRF were submitted to the Royal British Columbia Museum (RBCM) after sequencing. Catalogue numbers are provided where applicable.

Species	Host	Location	12S (464 bp)	*cox1* (904 bp)	*cox2* (506 bp)	28S (933 bp)	ITS (756 bp)	*ard1* (637 bp)
***B. laevis* VI** RBCM 022-00021-001	*Marmota vancouverensis*	VI, CAN	**ON994381**	**ON982731**	**ON988167**	**ON994376**	**ON982744**	**ON988164**
***B. laevis* AK** UAM 130585	*M. monax*	Alaska, USA	**ON994382**	**ON982732**	**ON988168**	**ON994377**	**ON982755**	**ON988165**
***B. laevis* ID1** MSBP 24571	*Urocitellus columbianus*	Idaho, USA	**ON994383**	**ON982733**	**ON988169**			
***B. laevis* ID2** MSBP 24572	*U. columbianus*	Idaho, USA	**ON994384**	**ON982734**	**ON988170**			**ON988166**
***B. laevis* ID3** MSBP 24594	*U. columbianus*	Idaho, USA	**ON994385**		**ON988171**	**ON994378**		
***B. columnaris* CT**	*Mephitis mephitis*	Connecticut, USA	MG937785	MH795147	MH469662	MG927772	MH030594	MH900134
***B. columnaris* IL**	*M. mephitis*	Illinois, USA	MG937786	MH795148	MH469663	MG927773	MH030595	MH900135
***B. procyonis* CT**	*Procyon lotor*	Connecticut, USA	MG937787	MH795149	MH469664	MG927774	MH030596	MH900136
***B. procyonis* CA**	*P. lotor*	California, USA	MG937788	MH795150	MH469665	MG927775	MH030597	MH900137
***B. devosi***	*Pekania pennanti*	Ontario CAN	MG937789	MH795151	MH469666	MG927776	MH030598	MH900138
***B. schroederi***	*Ailuropoda melanoleuca*	Sichuan, CHN	MG937790	MH795152	MH469667	MG927777	MH030599	MH900139
***B. ailuri***	*Ailuris fulgens*	Sichuan, CHN	MG937791	MH795153	MH469668	MG927778	MH030600	MH900140
***B. transfuga* ALB**	*Ursus arctos*	Alberta, CAN	MG937792	MH795154	MH469669	MG927779	MH030601	MH900141
***B. transfuga* WV**	*U. americanus*	West Virginia, USA	MG937793	MH795155	MH469670	MG927780	MH030602	MH900142
***B. tasmaniensis***	*Sarcophilus harrisii*	Tasmania, AUS	MG937794	MH795156	MH469671	MG927781	MH030603	MH900143
***Ascaris suum***	*Sus scrofa domesticus*	Louisiana or Michigan, USA	MG937795	MH795157	MH469672	MG927782	MH030604	MH900144
***Parascaris equorum***	*Equus ferus caballus*	Louisiana, USA	MG937796	MH795158	MH469673	MG927783	MH030605	MH900145
***Toxascaris leonina***	*Vulpes vulpes*	South Dakota, USA	MG937797	MH795159	MH469674	MG927784	MH030606	MH900146

### 2.2 PCR amplification and sequencing

Genes were selected for analysis based on availability of GenBank accessions from related species. Six genes had previously been used to identify relationships among seven *Baylisascaris* species (Camp et al., 2018) (refer to Table 1 for species details). These included three mitochondrial genes (12S rDNA, cytochrome oxidase subunits 1 and 2 (*cox1* and *cox2*)) and three nuclear genes (large-subunit rDNA (28S), internal transcribed spacers 1–2 (ITS), and the exon-primed intron-crossing (EPIC) locus alcohol/ribitol dehydrogenase (*ard1*)). For *Diandrya*, we selected *cox1* and ITS1 because these genes have previously been sequenced for *D. composita* and related species by Wickström et al. (2005) (Table 2). We designed new primers for several genes in Primer3 v2.3.7 (Untergasser et al., 2012) based on congeneric DNA sequence alignments to improve amplification success (Table 1, Table 2, Table 3).

**Table 2:** GenBank accession numbers for *Diandrya* and outgroup sequences included in analysis. The length of each alignment is listed in nucleotide base pairs. Catalogue numbers for *D. vancouverensis* specimens submitted to the Royal British Columbia Museum (RBCM) are below each sample name.

Species	Host	Location	*cox1* (568 bp)	ITS1 (863 bp)
***D. vancouverensis* 1** RBCM 022-00022-001	*Marmota vancouverensis*	VI, CAN	**ON982735**	**ON982764**
***D. vancouverensis* 2** RBCM 022-00026-001	*M. vancouverensis*	VI, CAN	**ON982736**	**ON982765**
***Diandrya composita* 1**	*M. caligata*	Alaska, USA	AY181550	
***D. composita* 2**	*M. broweri*	Alaska, USA	AY568212	
***D. composita* 3**	*M. caligata*	Alaska, USA	AY181551	AY752649
***Eurotaenia gracilis***	*Microtus agrestis*	Luhanka, FIN	AY395633	AY752656
***Douthittia nordenskioeldi***	*Dicrostonyx* sp.	Victoria Island, CAN	AY568204	AF314411
***Hymenolepisdiminuta***	*Rattus* sp.	Perth, AUS	NC_002767	AF461125
***Andrya cuniculi***	*Oryctolagus cuniculus*	Unspecified	AY189957	AF314409

Each gene was amplified via polymerase chain reaction (PCR) containing 12.5 μl of 2x TopTaq Master Mix (Qiagen, Toronto, Ontario, Canada), 200 μM deoxynucleotide triphosphates, 0.4 μM of forward and reverse primer, 3 mM total MgCl_2_, 3 μl (∼200–600 ng) of template genomic DNA (gDNA), and ribonuclease-free water, to a final volume of 25 μl. We increased the concentration of the forward primer to 0.8 μM for *cox2* following Nadler and Hudspeth (2000) and increased the amount of gDNA and total MgCl_2_ (3.5–5 mM) when our extractions yielded lower concentrations of DNA. Table 3 lists all primers and annealing temperatures (Ta) used for amplification and sequencing. Cycling parameters for *Baylisascaris* 12S, *cox1*, *cox2*, and ITS, and *Diandrya* ITS1, comprised 4 min denaturation at 94 °C, followed by 35 cycles of 30 s at 94 °C, 30 s at Ta, 70 s at 72 °C, and a final extension for 7 min at 72 °C. Annealing time was increased to 45 s for *Baylisascaris ard1* and 28S, as well as *Diandrya cox1*, while the number of cycles was increased to 45 for extractions with low concentrations of DNA. Amplicons were Sanger sequenced in both directions at the University of Alberta's Molecular Biology Services Unit. Consensus sequences, for each sample at each gene, were extracted from overlapping forward and reverse sequences after trimming low quality ends and primer regions, using Geneious v10.2.6 (Kearse et al., 2012). For *Diandrya* ITS1, consensus sequences were extracted from alignments produced with both primer pairs listed in Table 3, although the primer pair DvITS1F-2 and DvITS1R-2 generally reproduced the consensus sequence. Newly generated GenBank accession numbers for *B. laevis* and *D. vancouverensis* are available in Table 1, Table 2

**Table 3:** Primers used in PCR amplification. F/R indicates forward or reverse direction of primer. Annealing temperate is abbreviated as Ta. Product sizes are reported in base-pairs (bp).

Locus	Primer	F/R	Sequence (5′-3′)	Ta (°C)	Size (bp)	Reference
*Baylisascaris*						
12S	505	F	GTTCCAGAATAATCGGCTAGAC	50	493	Nadler et al. (2006)
	506	R	TCTACTTTACTACAACTTACTCCCC			
*cox1*	Blcox1F	F	TGGGTTGTGGTACTAGTTGGA	56	912	This study
	Blcox1R	R	AGACCCATAAGAAGCCACAA			
*cox2*	211	F	TTTTCTAGTTATATAGATTGRTTTYAT	50	582	Nadler and Hudspeth (2000)
	210	R	CACCAACTCTTAAAATTATC			
28S	Bl28SF	F	AGTAACTGCGAGTGAACGGG	60	943	This study
	Bl28SR	R	TCGCCCCTATACCCAAGTCA			
ITS	dp617	F	CTCCGAACGTGCATAAGCACC	58	829	Camp et al. (2018)
	94	R	TTAGTTTCTTTTCCTCCGCT			
*ard1*	Blard1F	F	TATGGGCCAGAGAGGTGTCA	58	703	This study
	Blard1R	R	CAGCGTCATACCGGCAATTG			
*Diandrya*						
*cox1*	COX-F	F	GATGTTTTCTTTACATTTATCTGGTG	51	640	Haukisalmi et al. (2004)
	COX-R	R	GCCACCACAAATCAAGTATC			
ITS1	DvITS1F-1	F	GTAACAAGGTAGCTGTAGG	56	642	This study
	DvITS1R-1	R	GCGATTCACATTAATTCACA			
	DvITS1F-2	F	AAGGTAGCTGTAGGTGAACC	56	664	This study
	DvITS1R-2	R	TGTCGATGTTCAAAGCAGTC			

### 2.3 Alignment and phylogenetic analyses

Phylogenetic analyses were conducted for individual genes as well as concatenated sequence datasets of mitochondrial, nuclear, and/or combined loci, where sequences were available. Only *Baylisascaris* specimens from Idaho failed to produce sequence data at every gene, and thus were excluded from certain concatenated and gene-specific analyses. Outgroup taxa for *Baylisascaris* were *Ascaris suum*, *Parascaris equorum*, and *Toxascaris leonina*, following Camp et al. (2018).

*Hymenolepis diminuta* was selected as the outgroup taxon for *Diandrya* analyses, following Wickström et al.’s (2005) molecular phylogeny of anoplocephaline cestodes. *Douthittia nordenskioeldi, Andrya cuniculi*, and *Eurotaenia gracilis* were included as ingroup species to improve the resolution of our *Diandrya* phylogeny. Wickström et al. (2005) placed *D. nordenskioeldi* as sister to *Diandrya composita*, whereas *A. cuniculi* was considered sister to *D. composita* prior to their analysis. *Eurotaenia gracilis* returned the best overall hits for *Diandrya vancouverensis* at *cox1* and ITS1 in NCBI's nucleotide database using their basic local alignment search tool (Johnson et al., 2008; Sayers et al., 2022).

Nucleotide sequences from *B. laevis* and *D. vancouverensis* were aligned with congeneric and outgroup sequences obtained from GenBank (Table 1, Table 2). Non-protein coding genes (12S, 28S, and ITS), the intron-crossing locus *ard1*, and concatenated sequences were aligned with ProbAlign v1.4 (Roshan and Livesay, 2006) using default parameters. Nucleotide sequences of protein coding genes *cox1* and *cox2* were aligned in Geneious using default parameters. To control for errors arising from alignment, nucleotide sequences of *cox1* and *cox2* were translated *in silico* with ExPasy (http://web.expasy.org/translate/) and the resulting amino acid sequences were aligned in Geneious. These amino acid alignments were uploaded as a scaffold for alignment of *cox1* and *cox2* nucleotide sequences in RevTrans v2.0 (Wernersson and Pedersen, 2003). The resulting alignments were identical to nucleotide alignments produced by Geneious. In addition, nucleotide alignments for all genes were tested for ambiguously aligned sites using Gblocks v0.91b (Castresana, 2000) with default parameters in the Phylogeny. fr web server (Dereeper et al., 2008).

Similar to Wickström et al. (2005) and Camp et al. (2018), we used both maximum parsimony (MP) and Bayesian inference (BI) to reconstruct evolutionary relationships. MP selects optimal trees in terms of the least number of substitutions required to account for the observed variation (Steel and Penny, 2000), whereas BI populates trees by stochastic, stepwise base substitution and evaluates their posterior probabilities against prior assumptions, i.e., pre-determined models of evolution based on large sequence datasets (Lartillot, 2020). For both MP and BI, we performed four rounds of analysis to reconstruct 1) individual single gene relationships (single gene trees), 2) mitochondrial relationships (mt-trees), 3) nuclear relationships (nr-trees), and 4) combined mitochondrial and nuclear relationships (combined trees).

MP analyses were performed in PAUP* 4.0a (Swofford and Sullivan, 2012) using a commonly applied heuristic search strategy (e.g., Catalan et al., 1997; Steane et al., 1999; Janes et al., 2010). Constant and autapomorphic sites were excluded (Supplementary Table S1). For each gene or concatenated dataset, we performed three stepwise heuristic searches allowing for multiple trees: 1. Closest addition sequence; 2. Random addition sequence with 5000 replicates; and 3. Constrained random addition sequence with 5000 replicates using the strict consensus of the optimal search from previous steps. Bootstrapping, using 1000 replicates of fast stepwise addition, was performed on the strict consensus obtained from step 3. All searches were performed using tree-bisection reconnection (TBR) branch swapping. Support for nodes is expressed as bootstrap values above branches.

The mitochondrial protein coding genes *cox1* and *cox2* were partitioned as individual genes, to capture differences in the evolution of each gene or codon position, using the Greedy algorithm in PartitionFinder v2.1.1 (Lanfear et al., 2017) after AIC and likelihood model selection with PhyML 3.0 (Guindon et al., 2010). For all other genes, best-fit evolutionary models were selected based on AIC values using MrModeltest v2.4 (Nylander, 2004) (Supplementary Table S2). Partitions were applied to concatenated nuclear, mitochondrial, and combined datasets to apply the appropriate model to each gene or codon position. Concatenated and individual gene datasets were used in BI analyses with the MrBayes v3.2.6 (Ronquist et al., 2012) plugin for Geneious. BI analyses were run using the selected models with the following settings: four Markov Chain Monte Carlo (MCMC) runs of 15 million generations, subsampling every 5000 generations, and 25% burn-in. Traces and parameter estimates were viewed in Geneious to ensure estimated sample sizes (ESS) > 400 for each dataset. Support for nodes is expressed as posterior probabilities (BPP). Both MP and BI consensus trees were visualized as phylograms in Geneious.

## 3 Results

### 3.1 Sequencing success

All *Baylisascaris* amplicons from VI and Alaska produced high quality consensus sequences of sufficient length for analysis. Samples from Idaho were inconsistent in producing useable sequences (Table 1) therefore, Idaho ITS sequences were excluded from further analyses. All other single gene *Baylisascaris* datasets contained sequences from at least one Idaho specimen, and datasets for 12S and *cox2* contained sequences from all *Baylisascaris* specimens listed in Table 1. Amplicons from both *Diandrya* specimens produced high quality consensus sequences at *cox1* and ITS1 (Table 2). The proportion of ambiguously aligned sites identified by Gblocks for non-protein coding genes ranged from 1% for *B. laevis* 28S to 58% for *D. vancouverensis* ITS1, and are reported in Supplementary Table S3. No such sites were found for alignments of *cox1* or *cox2*.

### *3.2* Molecular phylogenetics of Baylisascaris laevis

**Fig. 2:**
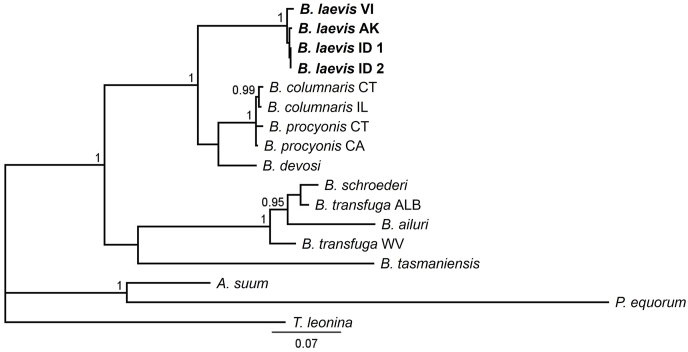
Bayesian consensus phylogram based on *Baylisascaris* and outgroup alignments of concatenated mitochondrial sequences (12S, *cox1*, and *cox2*) from GenBank and this study (*B. laevis*). Branch labels represent Bayesian posterior probabilities. Branch lengths are scaled to expected number of substitutions per site. Abbreviations refer to sampling sites (AK = Alaska, ID = Idaho, CT = Connecticut; IL = Illinois; CA = California; WV = West Virginia; ALB = Alberta). See also Table 1.

**Fig. 3:**
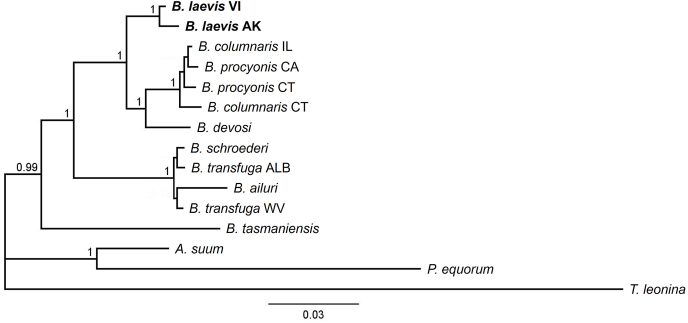
Bayesian consensus phylogram based on *Baylisascaris* and outgroup alignments of concatenated nuclear sequences (28S, ITS, and *ard1*) from GenBank and this study (*B. laevis*). Branch labels represent Bayesian posterior probabilities. Branch lengths are scaled to expected number of substitutions per site. Abbreviations refer to sampling sites (AK = Alaska, ID = Idaho, CT = Connecticut; IL = Illinois; CA = California; WV = West Virginia; ALB = Alberta). See also Table 1.

**Fig. 4:**
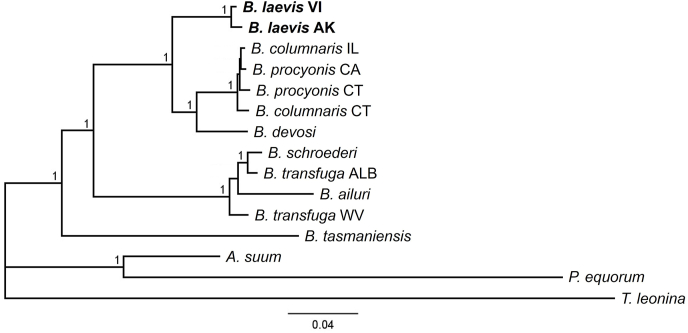
Bayesian consensus phylogram based on *Baylisascaris* and outgroup alignments of concatenated mitochondrial (12S, *cox1*, and *cox2*) and nuclear sequences (28S, ITS, and *ard1*) from GenBank and this study (*B. laevis*). Branch labels represent Bayesian posterior probabilities. Branch lengths are scaled to expected number of substitutions per site. Abbreviations refer to sampling sites (AK = Alaska, ID = Idaho, CT = Connecticut; IL = Illinois; CA = California; WV = West Virginia; ALB = Alberta). See also Table 1.

All BI and MP reconstructions across mt-trees, nr-trees, and combined trees consistently resolved *B. laevis* as a monophyletic group with high support (Fig. 2, Fig. 3, Fig. 4, Supplementary Figs. S1–S3). BI and MP single gene trees (Figs. S4–S15) reflected this relationship. All mt-trees, nr-trees, and combined trees supported the two clades resolved by Camp et al. (2018), splitting *B. columnaris*, *B. procyonis*, and *B. devosi* from *B. schroederi*, *B. transfuga*, and *B. ailuri* (Fig. 2, Fig. 3, Fig. 4, S1–S3), although most trees placed *B. tasmaniensis* as sister to all other *Baylisascaris* species, apart from the BI mt-tree (Fig. 2). The mt-trees, nr-trees, and combined trees showed strong support for *B. laevis* as sister to the clade containing *B. columnaris*, *B. procyonis*, and *B. devosi* (Fig. 2, Fig. 3, Fig. 4, S1–S3). Most single gene trees supported this relationship, with the following exceptions: 1) the 12S MP and BI trees had an alternate branching pattern between *B. laevis* and *B. devosi*; 2) the *cox1* MP tree placed *B. laevis* as sister to all *Baylisascaris*; and 3) the *cox2* BI tree, and 28S trees from MP and BI, presented unresolved polytomies between *B. laevis* and the other species in this clade (Supplementary Figs. S3 and S4, S6, and S9–S11 respectively).

The mean pairwise sequence divergence (*Mean ± SD*) for *B. laevis* was 0.96 ± 0.70% between VI and Alaska; 1.12 ± 1.01% between VI and Idaho; and 1.12 ± 0.78% between VI and all mainland sequences (Table 4). The low divergence between VI and Alaska was consistent with topologies reconstructed in the BI and MP trees for individual nuclear genes, which showed VI and Alaska samples as sister taxa (Supplementary Figs. S10–S15). The trees for individual mitochondrial genes, and the concatenated mt-trees, were unable to resolve the relationships among *B. laevis* samples or placed the VI sample as sister to all other *B. laevis* samples (Fig. 2, S1, and S4–S9).

**Table 4:** Mean pairwise percent sequence divergence across genes for *Baylisascaris laevis* from Vancouver Island (VI), Alaska (AK), Idaho (ID), and combined mainland (ML) sequences. Genes for which no paired sequences are available are denoted ‘-‘.

Locus	VI-AK	VI-ID	VI-ML	AK-ID	ID - ID
12S	0.44	0.22	0.33	0.22	0.00
28S	0.11	0.75	0.43	0.86	–
*ard1*	1.25	2.86	2.05	3.39	–
*cox1*	0.66	0.77	0.72	0.33	0.00
*cox2*	1.19	0.99	1.09	0.20	0.00
ITS	2.08	–	2.08	–	–
*Mean* ± *SD*	0.96 ± 0.70	1.12 ± 1.01	1.12 ± 0.78	1.00 ± 1.36	–

### *3.3* Molecular phylogenetics of Diandrya vancouverensis

**Fig. 5:**
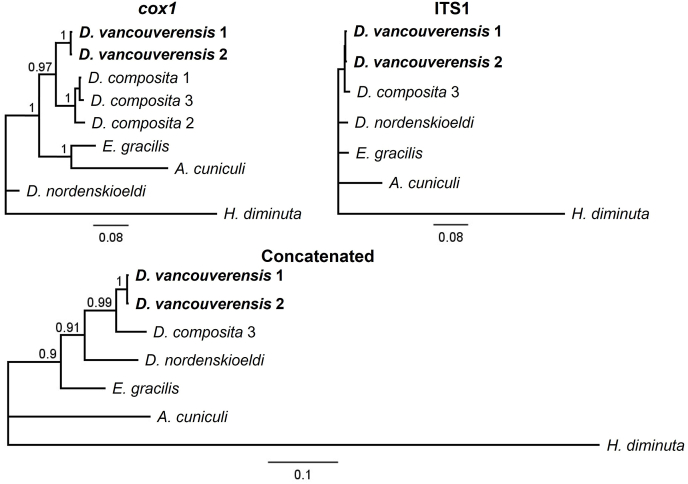
Bayesian consensus tree from *Diandrya*, related genera, and outgroup alignments of *cox1*, ITS1, and concatenated sequences from GenBank and this study (*D. vancouverensis*). Branch labels represent Bayesian posterior probabilities. Branch lengths are scaled to expected number of substitutions per site. See also Table 2.

BI trees for *cox1* and combined genes placed *D. vancouverensis* as a monophyletic clade with high BPP support, whereas BPP support at ITS1 alone was less than 0.90 (Fig. 5). All MP trees strongly supported the monophyletic clade of *D. vancouverensis* (Supplementary Fig. S16). Mean pairwise sequence divergence between *D. vancouverensis* and *D. composita* was 9.06% (7.69% at *cox1* and 10.44% at ITS1), and the two VI samples had identical sequences at both genes.

## 4 Discussion

*Baylisascaris laevis* and *Diandrya vancouverensis* represent two parasite species that have previously been classified solely based on morphology and ecology (Sprent, 1968; Mace and Shepard, 1981). Whereas *B. laevis* is common among ground squirrels and marmots across North America, *D. vancouverensis* has been described as endemic to the VIM. Until now, the extent to which their obligate association with this rare, island host has contributed to their genetic divergence and potential conservation concern was unclear. We reconstructed the phylogenetic relationships of both parasite species and estimated the degree of divergence between VI and mainland populations using new sequence data. Our results support the species designation of *D. vancouverensis* and present evidence for divergence of *B. laevis* on VI.

### 4.1 Systematics of Baylisascaris laevis

Camp et al. (2018) proposed two clades in *Baylisascaris* based on reconstructions across eight genes for seven of the 11 recognized species in the genus. Our reconstructions with additional samples and species supported these two clades; however, our analyses generally placed *B. tasmaniensis* as sister to all other *Baylisascaris* species, except in the *cox1* and *cox2* trees (Figs. S6, S7, and S9), the BI 28S tree (similar to the BI 28S tree from Camp et al. (2018; Fig. S11)), and the MP and BI *ard1* trees (Figs. S12–S13). The proportion of unambiguous sites selected by Gblocks in our *Baylisascaris* alignments (Supplementary Table S3) were comparable to the proportion of characters retained by Camp et al. (2018; Supplementary Data) in datasets filtered based on a 60% posterior probability threshold with ProAlign (Löytynoja and Milinkovitch, 2003).

The topology of ingroup species (excluding *B. laevis*) in our BI mt-tree matched Camp et al. (2018), although *B. procyonis* from California and Connecticut were placed in a polytomy that was sister to *B. columnaris* (Fig. 2). Similarly, our BI nr-tree showed an alternate topology for *Baylisascaris* spp. in the clade with ursid and ailurid hosts and placed *B. tasmaniensis* as sister to all other *Baylisascaris* (Fig. 3). Our BI combined tree followed our mt-tree topology for the ursid and ailurid clade, but otherwise reflected our nr-tree arrangement of *Baylisascaris* spp. (Fig. 4). These differences may reflect the fact that our analyses excluded two nuclear genes included in the analysis by Camp et al. (2018).

Our analyses placed *B. laevis* in the clade with (but also sister to) species that parasitize skunks, raccoons, and gulonine mustelids (*B. columnaris*, *B. procyonis*, and *B. devosi*, respectively). This relationship confirms the morphological grouping assigned by Sprent (1968) and constitutes the first molecular phylogenetic placement of *B. laevis*. It has previously been suggested that the direct life cycle of *B. laevis* is the result of a capture event in its evolution, during which an ancestral form with a carnivoran host was able to develop to adulthood within its rodent intermediate host (Anderson, 2000). The position of *B. laevis* as sister to one clade, but not the other, suggests that this change likely occurred after the common ancestors of each clade diverged.

The pairwise sequence divergence between the VI and mainland samples of *B. laevis* ranged from 0.11 to 2.86% (Table 4), with the greatest divergence observed between VI and Idaho at *ard1*. However, variation between VI and Idaho was surpassed by variation between Idaho and Alaska at the same locus (3.39%). Locus *ard1* is an EPIC marker selected to capture variability between closely related species (Camp et al., 2018); it is highly variable by design, and thus likely not representative of mean divergence for the purpose of species delimitation. ITS and *cox1*, on the other hand, have been used to estimate interspecific divergence and prospect for cryptic species within nematode genera (Powers et al., 1997; Blouin, 2002).

Pérez Mata et al. (2016) used ITS to classify the novel species *Baylisascaris venezuelensis* and reported interspecific pairwise divergences of 2.8–10.0% among *B. venezuelensis, B. schroederi*, *B. transfuga*, and *B. procyonis*. Clearly, the extent of interspecific divergence within *Baylisascaris* encompasses a range of values depending on both the genes and species pairs examined. *Baylisascaris columnaris* and *B. procyonis* are considered distinct species with shallow genetic divergence (Franssen et al., 2013; Camp et al., 2018). In our analyses, these species formed a sister clade to *B. laevis*, and are thus a reasonable comparison for interspecific divergence values in closely related species.

The mean pairwise divergences between VI and mainland *B. laevis* at ITS and *cox1* (2.08 and 0.72%, respectively) were comparable to (or greater than) the divergences we calculated between *B. columnaris* and *B. procyonis* for the same genes in our alignment (1.12 and 0.88%, respectively). However, we note that Camp et al. (2018, Supplementary Data) reported mean divergences of 0.32 and 0.96% at ITS and *cox1*, respectively, between *B. columnaris* and *B. procyonis* in their alignments. These discrepancies may be due to differences in either the length of alignments (e.g., ITS sequences from Camp et al. (2018) were 889–975 bp long, whereas our alignment was truncated to 756 bp), or the algorithms used to construct them. Furthermore, Carlson et al. (2021) found *B. procyonis* had a maximum divergence of 1.6% at *cox1* when comparing populations on opposite sides of the continental divide.

The divergence of *B. laevis* between VI and the mainland may be within the range of interspecific divergences for *Baylisascaris* nematodes. However, our current data are insufficient to fully test our hypothesis of speciation for *B. laevis* on VI. More sophisticated analyses of species delimitation may be able to resolve this uncertainty. For example, Camp et al. (2018) included ITS and *cox1* in a species delimitation analysis using BP&P v3.3 (Bayesian Phylogenetics and Phylogeography; Yang, 2015). Further analyses must be informed by more extensive population sampling of *B. laevis* from both *M. vancouverensis* and mainland marmot populations (e.g., *M. caligata*), which we were unable to obtain here, in order to resolve the phylogeography and population genetic structure of *B. laevis* across its entire geographic and host ranges (Pérez-Ponce de León and Nadler, 2010). Further molecular analyses should ideally be complemented with detailed morphological comparisons of *B. laevis* from an equally broad range of hosts and localities in order to characterize its intraspecific variation within and among populations.

### 4.2 Systematics of Diandrya vancouverensis

Our combined and *cox1* trees separated *D. vancouverensis* and *D. composita* with high support (Fig. 5 and Fig. S16). Wickström et al. (2005) placed *D. composita* in a clade with *Paranoplocephala* spp. and Vilas et al. (2005) reported pairwise sequence divergences of 0.1–5.9% and 5.2–18.4% at ITS1 and *cox1*, respectively, among species pairs from *Paranoplocephala*. These values are comparable to the divergences between *D. vancouverensis* and *D. composita* for these genes (7.69 and 10.44%, respectively). Hence, this analysis supports the species classification of *D. vancouverensis* originally proposed based on its unique morphology (Mace and Shepard, 1981).

### 4.3 Factors influencing the divergence of helminths in the VIM

Host biogeography and metapopulation dynamics can play substantial roles in the phylogeography and evolution of parasitic species; however, the extent of co-phylogeny between parasites and their hosts is variable and inversely related to parasite characteristics such as virulence and host specificity (Gandon, 2002). Although they are associated with the same endemic insular host, *D. vancouverensis* appears to have diverged substantially more from its mainland counterpart than *B. laevis.* The observed disparity between the genetic divergences of these species may be explained by parasite-specific factors that affect their extent of co-speciation with their shared host.

Fossil evidence suggests that marmots have colonized VI multiple times during the Pleistocene, most recently following the last glacial maximum 12–13 Kya (Ward et al., 2003; Nagorsen and Cardini, 2009). Prolonged geographic isolation of the VIM, since its likely recolonization of VI, would provide an opportunity for extended divergence between marmot parasites on VI and those on the mainland. Molecular phylogenetic analyses from mitochondrial data have estimated divergence of the VIM from its sister species, the hoary marmot (*M. caligata*) between 0.4 and 1.2 million years ago (Mya) (Steppan et al., 2011). However, recent nuclear analyses have suggested earlier divergence followed by introgression of hoary mtDNA to the VIM 0.73 Mya (Kerhoulas et al., 2015, Kerhoulas, 2017). It is likely that shifting glacial barriers during the Pleistocene produced geographic isolation of these species, interspersed by periods of sympatry (Kerhoulas, 2017). Intermittent gene flow between VI and hoary marmots since their initial divergence may have provided transient opportunities for host-switching events, which would have disproportionately favoured gene flow among parasites with lower host specificity.

In general, the phylogenetic relationships within *Baylisascaris* do not closely mirror the biogeography or phylogeny of their host taxa, and colonization of novel hosts is thus expected to factor prominently in their diversification (Camp et al., 2018). For example, Zhou et al. (2013) reported an absence of population genetic structure in a metapopulation of *B. schroederi* from the giant panda (*Ailuropoda melanoleuca*), despite high levels of host differentiation across their fractured range. Furthermore, *Baylisascaris* spp. with hosts in the superfamily Musteloidea (gulonine mustelids, skunks, raccoons, and the red panda) are polyphyletic — *B. ailuri* from the red panda groups with species parasitizing members of the Ursidae. We note that *B. laevis* seems to have evolved after the split of the two major clades. This is intriguing as both clades generally represent species with hosts from Carnivora, whereas *B. laevis* is associated with hosts from Rodentia. This provides further evidence for a capture event during which *B. laevis* evolved the ability of direct transmission in an intermediate host (see section 4.1). These incongruences between the phylogeography of *Baylisascaris* species and their hosts suggests relatively low host specificity within the genus, which corresponds to low population genetic structure (Nadler, 1995).

Nematodes are dioecious and tend to evolve faster than other parasite lineages, despite the increased fecundity of monoecious species, such as cestodes (Anderson et al., 1998). *Baylisascaris laevis* is unique among its congeners in that it does not rely on intermediate hosts for transmission (Sapp et al., 2017). This feature of its life cycle likely facilitated its persistence on VI and may have conferred a dispersal advantage compared to *D. vancouverensis*, which, as an anoplocephalid cestode is expected to rely on a mite intermediate host for transmission (Denegri, 1993). Low host specificity in *Baylisascaris* could have contributed to increased gene flow among populations from different *Marmota* species during secondary contact events and may explain the weak divergence of *B. laevis* compared to *D. vancouverensis* (e.g., Haché et al., 2017). Each parasite will have had its own unique phylogeographic history involving colonization of a modern VIM or an ancestor, subsequent isolation, and episodic opportunities for gene flow, culminating in their present host-parasite assemblage (Hoberg and Brooks, 2008). The relative contributions of historical biogeography and life cycle characteristics to the divergence of these parasites alongside their shared host may be more clearly understood in light of their specific effects on marmot fitness, as well as direct analyses of their population genetic structure (e.g., Carlson et al., 2021).

### 4.4 Conservation implications

Antagonistic coevolution occurs when an adaptation in one symbiont (e.g., to increase exploitation by the parasite), elicits an evolutionary response by the other (e.g., to resist exploitation by the host) (Goater et al., 2014). Host-parasite competition can result in oscillatory fitness of parasite resistance genes, as described by the Red Queen hypothesis, when an adaptive response in the host becomes maladaptive as it is met by the reciprocal response from its parasite (Dybdahl and Lively, 1998). Under Red Queen dynamics, new adaptations become most effective when they are least common, which can drive genetic variation by selecting for rare phenotypes via negative frequency-dependent selection (Hamilton and Zuk, 1982). In terms of population fitness, the result of selection pressures on phenotypic diversity may depend on the existing genetic variance in the host population.

*Baylisascaris laevis* and *Diandrya* spp. have been associated with observable pathology in experimental and natural hosts, but neither have been directly implicated as cause of death (Babero, 1960; Raverty and Black, 2001). Detailed descriptions of their impact on VIM health will be a crucial next step in understanding their relevance within the VI ecosystem. The VIM has exceedingly low genetic diversity (Barrett et al., 2022) which may limit its future capacity for novel adaptation, making it more vulnerable to infection as its adaptive responses lag behind those of its parasites.

Parasite lineages currently represent a major source of biodiversity loss worldwide (Carlson et al., 2020). Until relatively recently, eradicating parasites during host species conservation has stunted research on their ecosystem relevance. Much like apex predators, parasites can serve important roles as trophic regulators (Dougherty et al., 2016). Discovery and delimitation of cryptic parasite species is a critical challenge in documenting parasite biodiversity which must be guided by rigorous systematic methodology (Pérez-Ponce de León and Nadler, 2010).

The critically endangered VIM is itself host to a cryptic community of endemic biodiversity. It has already been argued that *D. vancouverensis* must share the endangered status of the VIM (VIMRT, 2008). If future analyses find support to designate *B. laevis* on VI as a unique species, then this status should extend to both parasites. *Diandrya vancouverensis* and *B. laevis* represent the first examples of an otherwise uncharacterized community of endemic biodiversity within the VIM. Similarly, other symbionts may constitute cryptic species and are, in any case, intimately dependent on the fate of the VIM. Conservation efforts for the biotic community of the VIM must be informed by an appreciation of their systematics and unique associations with their host.

This study represents a crucial first step in characterizing the complex coevolutionary dynamics between the VIM and two of its endoparasites. Based on novel genetic sequences for each species, our analysis exceeds commonly accepted thresholds for species delimitation of *D. vancouverensis* and presents evidence for divergence of *B. laevis* on VI. Molecular phylogenies reconstructed using BI and MP algorithms reflect the relationships predicted by the morphology of each parasite species (Sprent 1968; Mace and Shepard, 1981; Camp et al., 2018). Unfortunately, we were unable to secure specimens of *B. laevis* from *M. caligata,* the sister species to *M. vancouverensis.* This comparison is a necessary next step in resolving the phylogeography of *B. laevis* on VI. Hopefully, further analyses of population genetic structure in *B. laevis* and *D. vancouverensis*, along with long-term monitoring of parasite burden in the VIM will contribute to an impactful appreciation of these relationships and will inform conservation efforts for all three species.

## Data availability

Parasite specimens collected from *M. vancouverensis* were submitted to the Royal British Columbia Museum and catalogue numbers are available in Table 1, Table 2, along with GenBank accession numbers for newly generated sequences.

## Declaration of competing interest

None.
