# Supplementary material for: Molecular phylogenetics and systematics of two enteric helminth parasites (Baylisascaris laevis and Diandrya vancouverensis) in the Vancouver Island marmot (Marmota vancouverensis)
Source: Int J Parasitol Parasites Wildl. 2022 Nov 17;19:301–10. doi: 10.1016/j.ijppaw.2022.11.006 (PMC9691466; doi:10.1016/j.ijppaw.2022.11.006)
Supplement: Multimedia component 1 [file mmc1.docx]

Molecular phylogenetics and systematics of two enteric helminth parasites (*Baylisascaris laevis* and *Diandrya vancouverensis*) in the Vancouver Island marmot (*Marmota vancouverensis*)

McIntyre A. Barrera, Jasmine K. Janes, Jamieson C. Gorrell

**SUPPLEMENTARY INFORMATION**

**Table S1**

Characters and statistics from maximum parsimony analyses, including concatenated mitochondrial (mit.) and nuclear (nuc.) loci. Constant and autapomorphic (apo.) characters were excluded. Rearrangements tried are listed for closest, random, and constrained (constr.) heuristic searches. Each search produced identical consistency (CI), homoplasy (HI), retention (RI), and rescaled consistency (RC) indices, number of trees retained, and best scores.

|  | Characters | | | | | Rearrangements | | | (Uninformative-excluded) | | | |  |  |
| --- | --- | --- | --- | --- | --- | --- | --- | --- | --- | --- | --- | --- | --- | --- |
| Locus | All | | Constant | Apo. | Inf. | Closest | Random | Constr. | CI | HI | RI | RC | Trees | Score |
| *Baylisascaris* | | | | | | | | | | | | | | |
| 12S | 464 | 341 | | 49 | 74 | 7736 | 710627 | 48110 | 0.57 | 0.43 | 0.72 | 0.41 | 4 | 162 |
| *cox1* | 904 | 702 | | 74 | 128 | 2452 | 2794383 | 0 | 0.52 | 0.48 | 0.65 | 0.34 | 1 | 311 |
| *cox2* | 506 | 386 | | 43 | 77 | 38152 | 1236902 | 12853 | 0.59 | 0.41 | 0.73 | 0.43 | 20 | 174 |
| 28S | 933 | 810 | | 71 | 52 | 2520 | 245404 | 48 | 0.74 | 0.26 | 0.84 | 0.62 | 2 | 77 |
| ITS | 756 | 530 | | 146 | 80 | 8632 | 82337 | 192 | 0.77 | 0.23 | 0.85 | 0.65 | 8 | 141 |
| *ard1* | 637 | 296 | | 200 | 141 | 2548 | 365587 | 23 | 0.68 | 0.32 | 0.75 | 0.51 | 2 | 277 |
| Mit. | 1897 | 1446 | | 168 | 283 | 3200 | 1050811 | 10476 | 0.54 | 0.46 | 0.67 | 0.36 | 2 | 669 |
| Nuc. | 2281 | 1615 | | 405 | 261 | 2208 | 440242 | 43 | 0.70 | 0.30 | 0.78 | 0.55 | 2 | 483 |
| All | 4199 | 3087 | | 570 | 542 | 3321 | 205540 | 4684 | 0.61 | 0.39 | 0.70 | 0.42 | 3 | 1151 |
| *Diandrya* | | | | | | | | | | | | | | |
| *cox1* | 568 | 387 | | 81 | 100 | 360 | 901 | 24 | 0.68 | 0.32 | 0.64 | 0.44 | 2 | 196 |
| ITS1 | 863 | 658 | | 177 | 28 | 72 | 6608 | 0 | 0.80 | 0.20 | 0.78 | 0.63 | 1 | 40 |
| All | 1431 | 1046 | | 271 | 114 | 72 | 18113 | 0 | 0.71 | 0.29 | 0.61 | 0.44 | 1 | 206 |

**Table S2**

Best-fit evolutionary models for Bayesian inference chosen for each locus or codon position (*cox1* and *cox2*).

| Locus | Model |
| --- | --- |
| *Baylisascaris* |  |
| 12S | HKY+G |
| 28S | HKY+G |
| *ard1* | GTR+G |
| Position 1 *cox1* | HKY+I |
| Position 2 *cox1* | GTR+G |
| Position 3 *cox1* | GTR+I |
| Position 1 *cox2* | GTR+I+G |
| Position 2 *cox2* | HKY+I |
| Position 3 *cox2* | HKY+G |
| ITS | HKY+I |
| *Diandrya* |  |
| Position 1 *cox1* | GTR+G |
| Position 2 *cox1* | GTR+G |
| Position 3 *cox1* | HKY |
| ITS1 | HKY+G |

**Table S3**

Percentage of ambiguously aligned sites for all genes included in this study. Identified using Gblocks v0.91 with default options.

| Alignment | Sequences | Length | Ambiguous sites |
| --- | --- | --- | --- |
| *Baylisascaris* **12S** | 18 | 464 | 32 (7%) |
| *Baylisascaris* ***cox1*** | 17 | 904 | 0 (0%) |
| *Baylisascaris* ***cox1*** | 18 | 506 | 0 (0%) |
| *Baylisascaris* **28S** | 16 | 933 | 7 (1%) |
| *Baylisascaris* ***ard1*** | 16 | 637 | 363 (57%) |
| *Baylisascaris* **ITS** | 15 | 756 | 263 (35%) |
| *Diandrya* ***cox1*** | 9 | 568 | 0 (0%) |
| *Diandrya* **ITS1** | 7 | 863 | 500 (58%) |

**
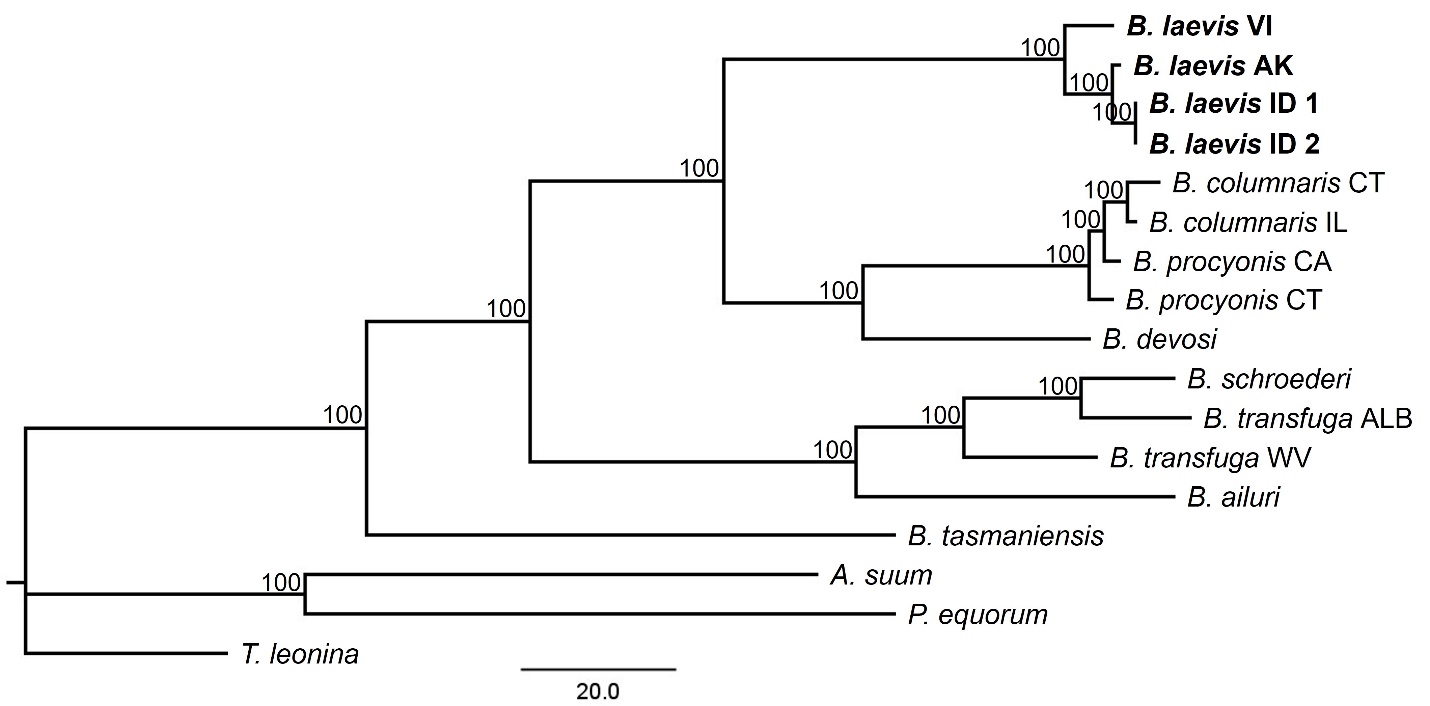
**

**Figure S1.** Phylogram based on strict consensus of maximum parsimony (MP) trees from *Baylisascaris* and outgroup alignments of concatenated mitochondrial loci (12S, *cox1*, and *cox2*). Branches are scaled to reflect character state substitutions. Bootstrap values above 0.90 are provided above each node.

**
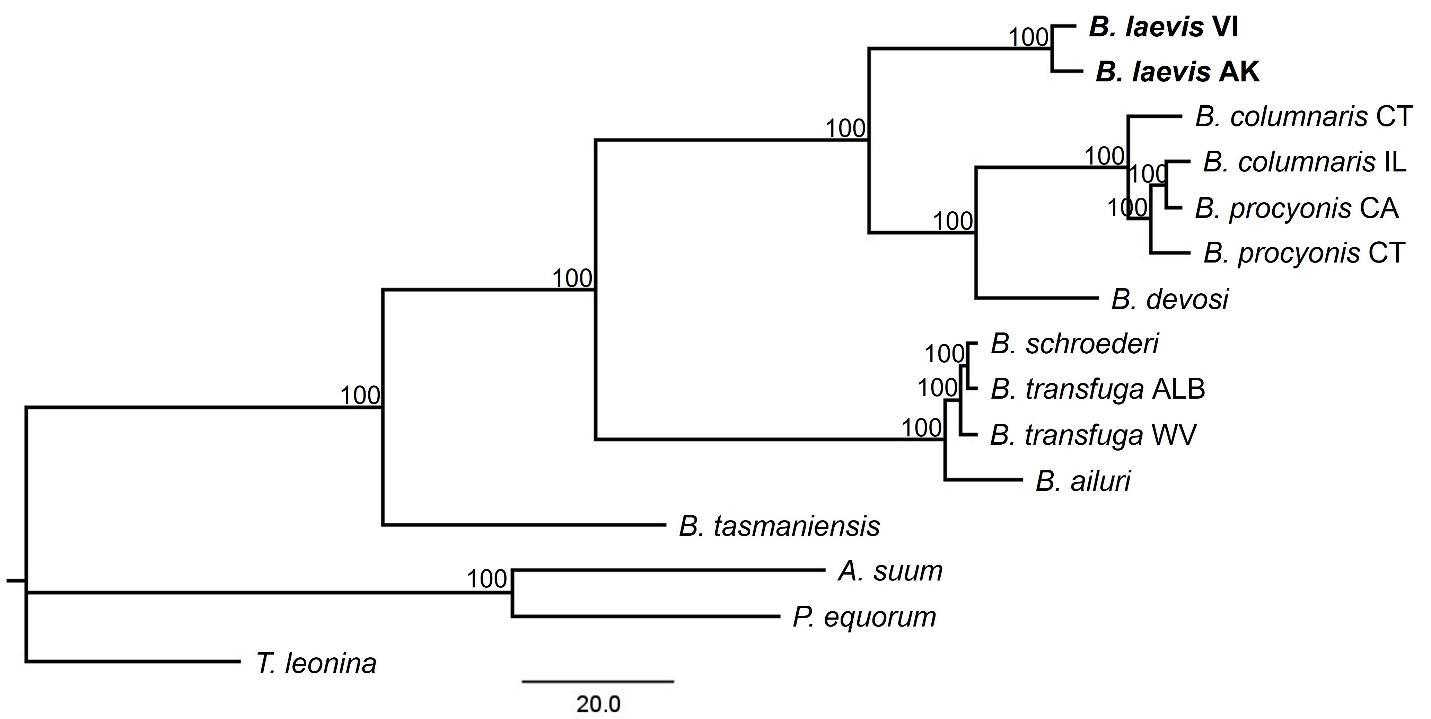
Figure S2.** Phylogram based on strict consensus of maximum parsimony (MP) trees from *Baylisascaris* and outgroup alignments of concatenated nuclear loci (28S, ITS, and *ard1*). Branches are scaled to reflect character state substitutions. Bootstrap values above 0.90 are provided above each node.


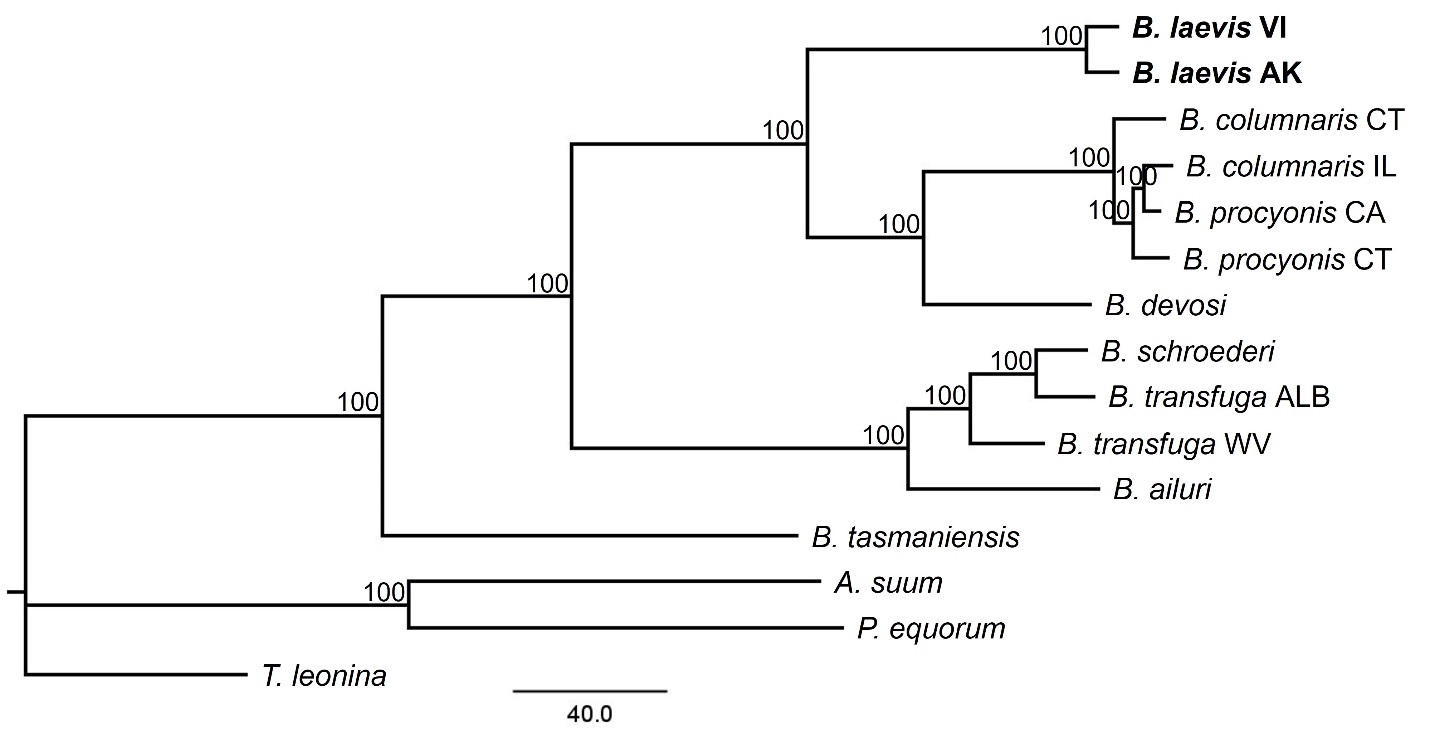


**Figure S3.** Phylogram based on strict consensus of maximum parsimony (MP) trees from *Baylisascaris* and outgroup alignments of concatenated mitochondrial and nuclear loci (12S, 28S, *cox1,* *cox2*, ITS, and *ard1*). Branches are scaled to reflect character state substitutions. Bootstrap values above 0.90 are provided above each node.


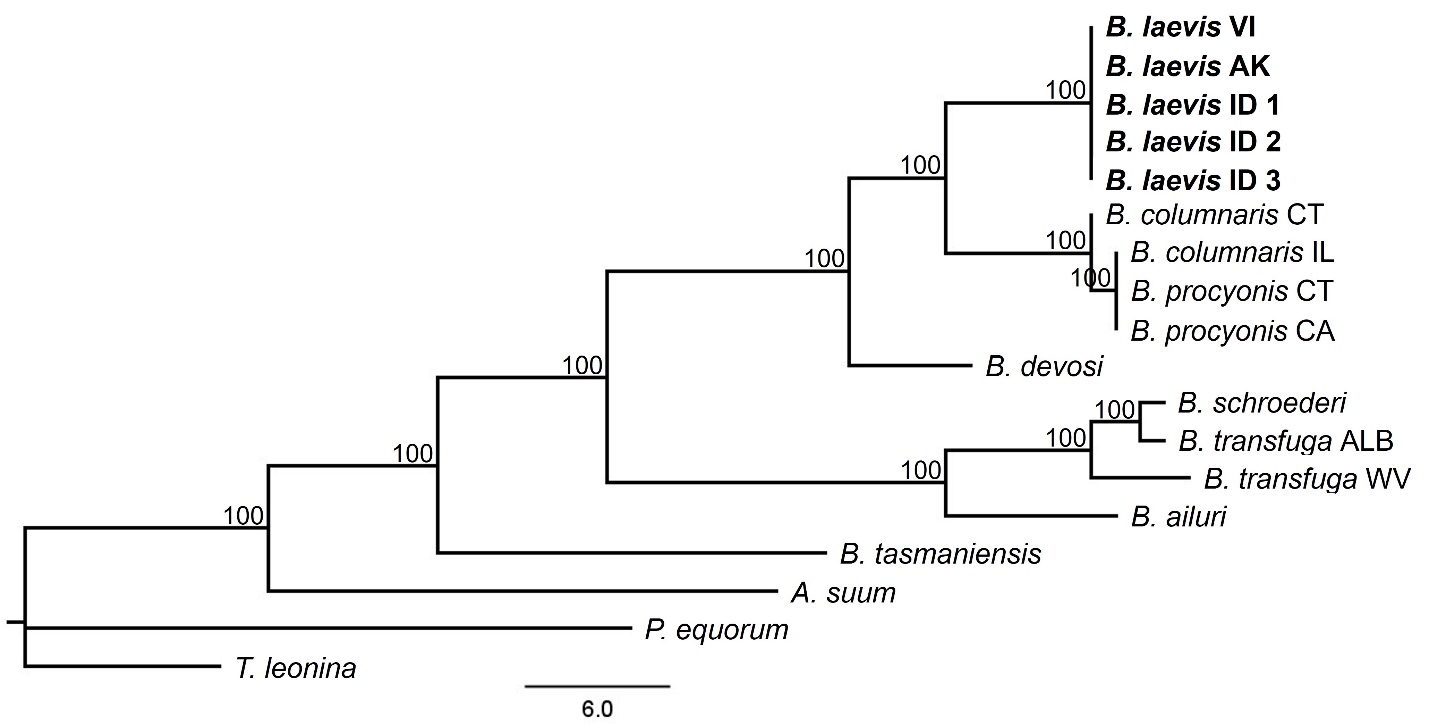


**Figure S4.** Phylogram based on strict consensus of maximum parsimony (MP) trees for *Baylisascaris* 12S. Branches are scaled to reflect character state substitutions. Bootstrap values above 0.90 are provided above each node.


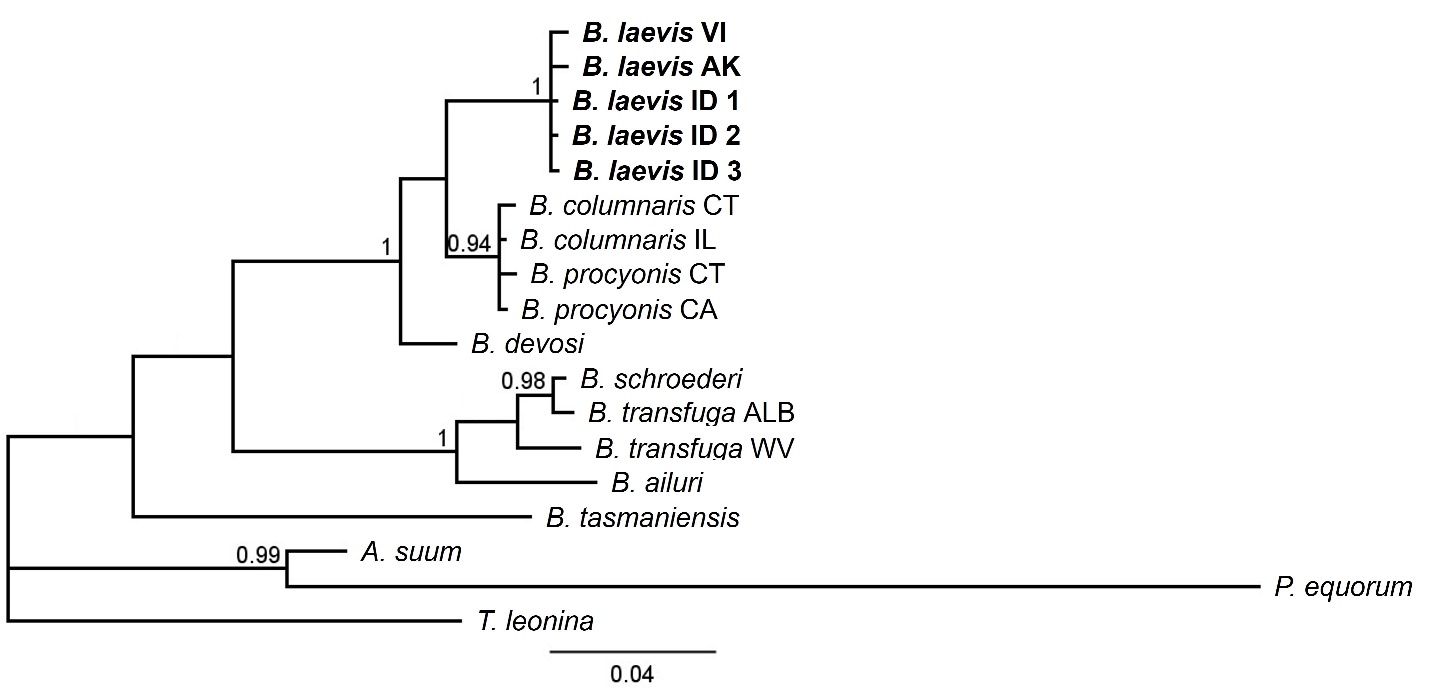


**Figure S5.** Bayesian consensus phylogram based on *Baylisascaris* and outgroup alignments of 12S. Branch labels represent Bayesian posterior probabilities. Branch lengths are scaled to expected number of substitutions per site.


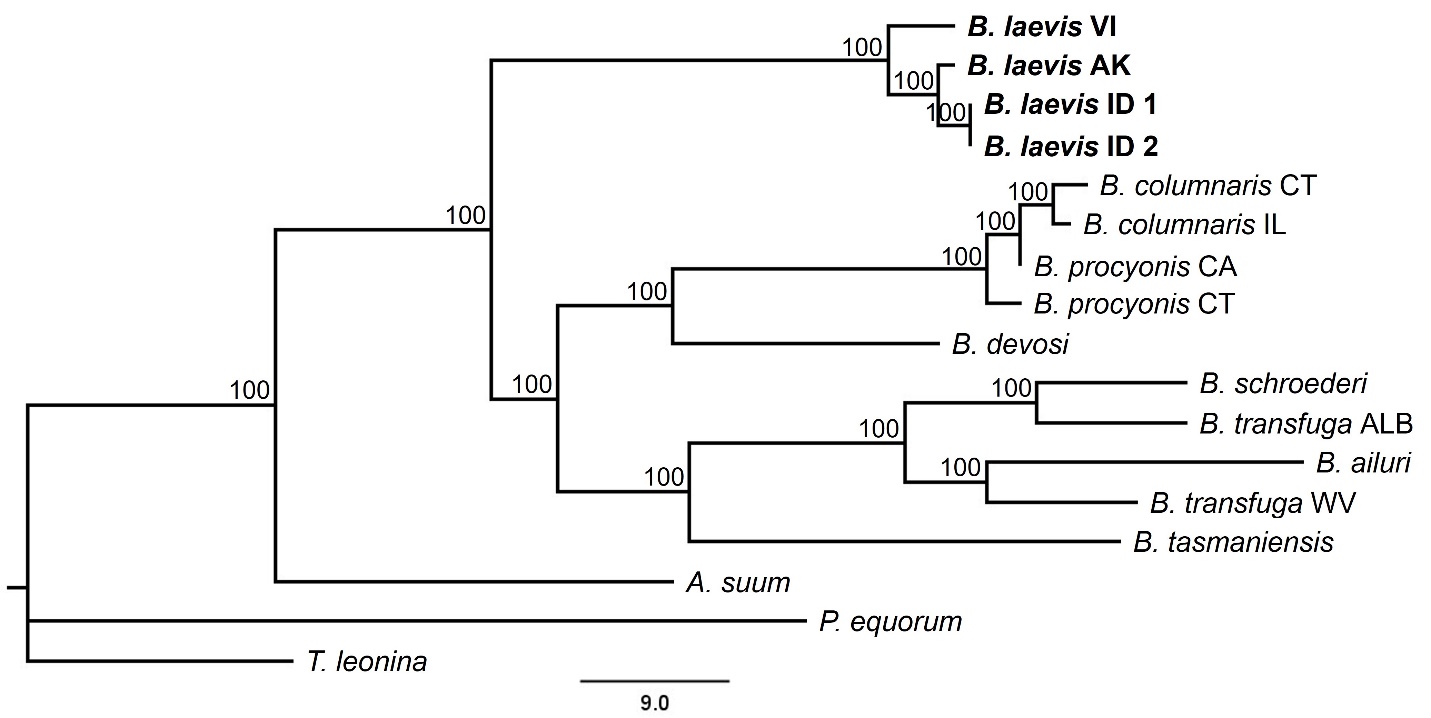


**Figure S6.** Phylogram based on strict consensus of maximum parsimony (MP) trees for *Baylisascaris cox1*. Branches are scaled to reflect character state substitutions. Bootstrap values above 0.90 are provided above each node.


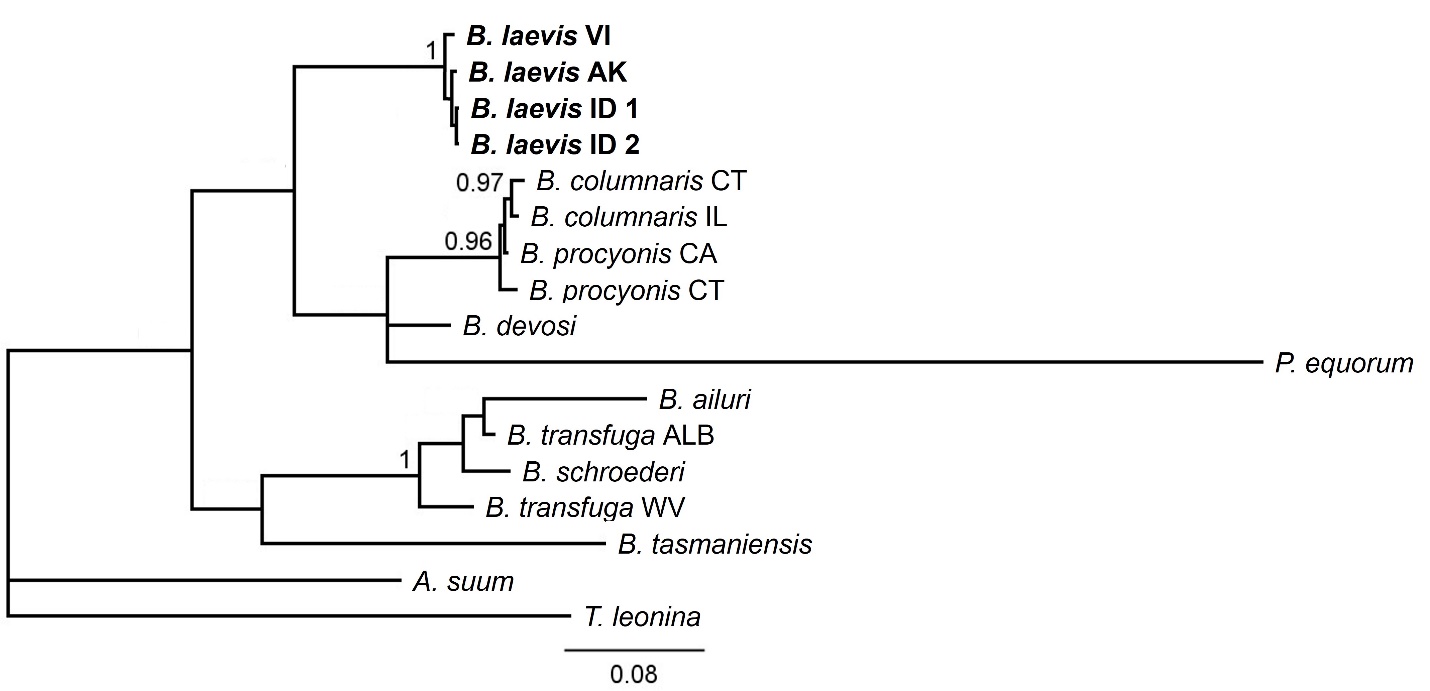


**Figure S7.** Bayesian consensus phylogram based on *Baylisascaris* and outgroup alignments of *cox1*. Branch labels represent Bayesian posterior probabilities. Branch lengths are scaled to expected number of substitutions per site.
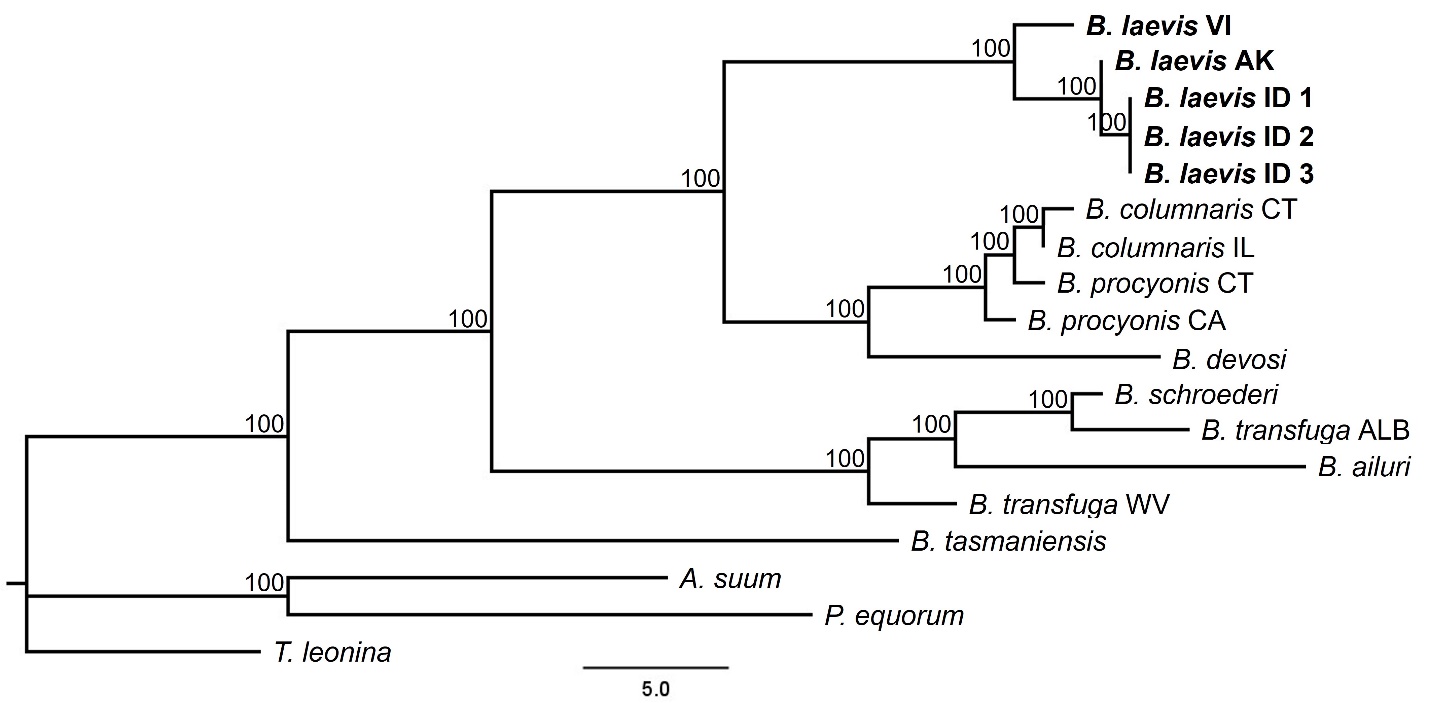


**Figure S8.** Phylogram based on strict consensus of maximum parsimony (MP) trees for *Baylisascaris cox2*. Branches are scaled to reflect character state substitutions. Bootstrap values above 0.90 are provided above each node.


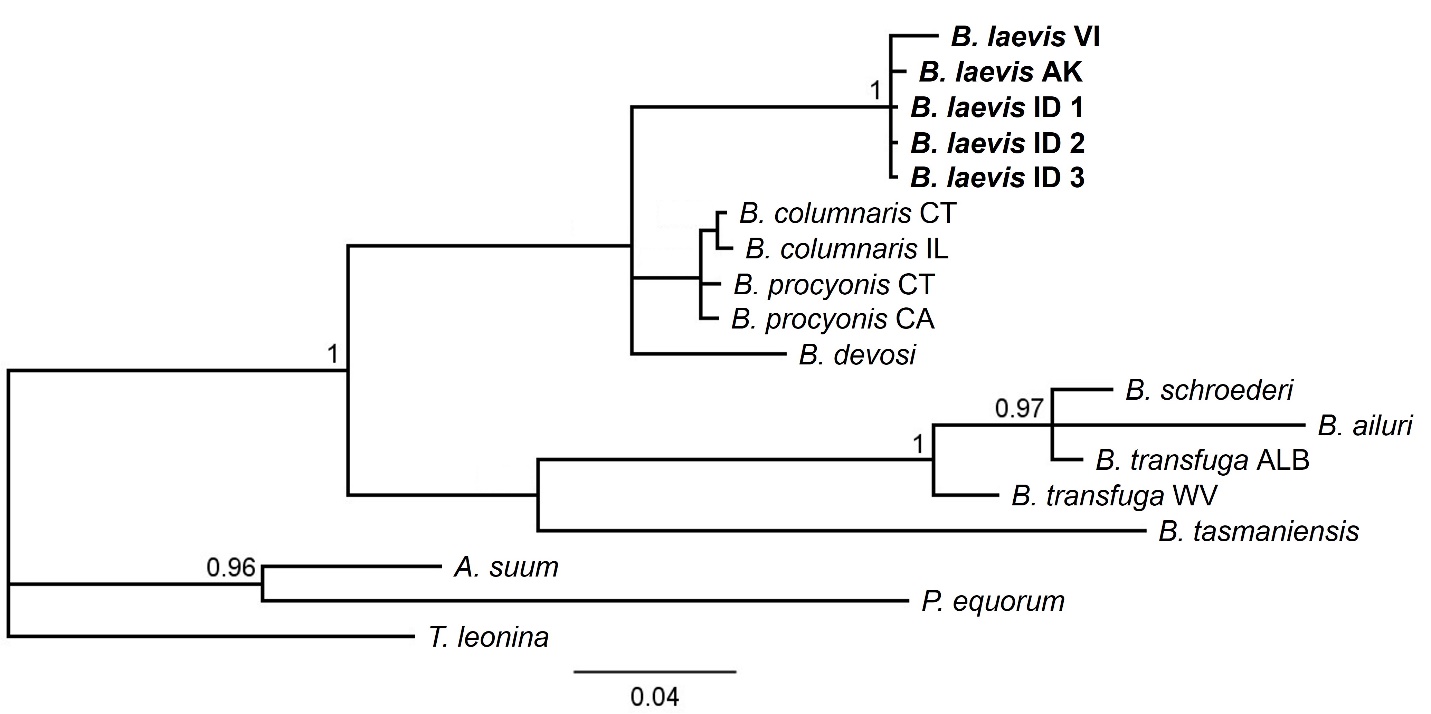


**Figure S9.** Bayesian consensus phylogram based on *Baylisascaris* and outgroup alignments of *cox2*. Branch labels represent Bayesian posterior probabilities. Branch lengths are scaled to expected number of substitutions per site.**
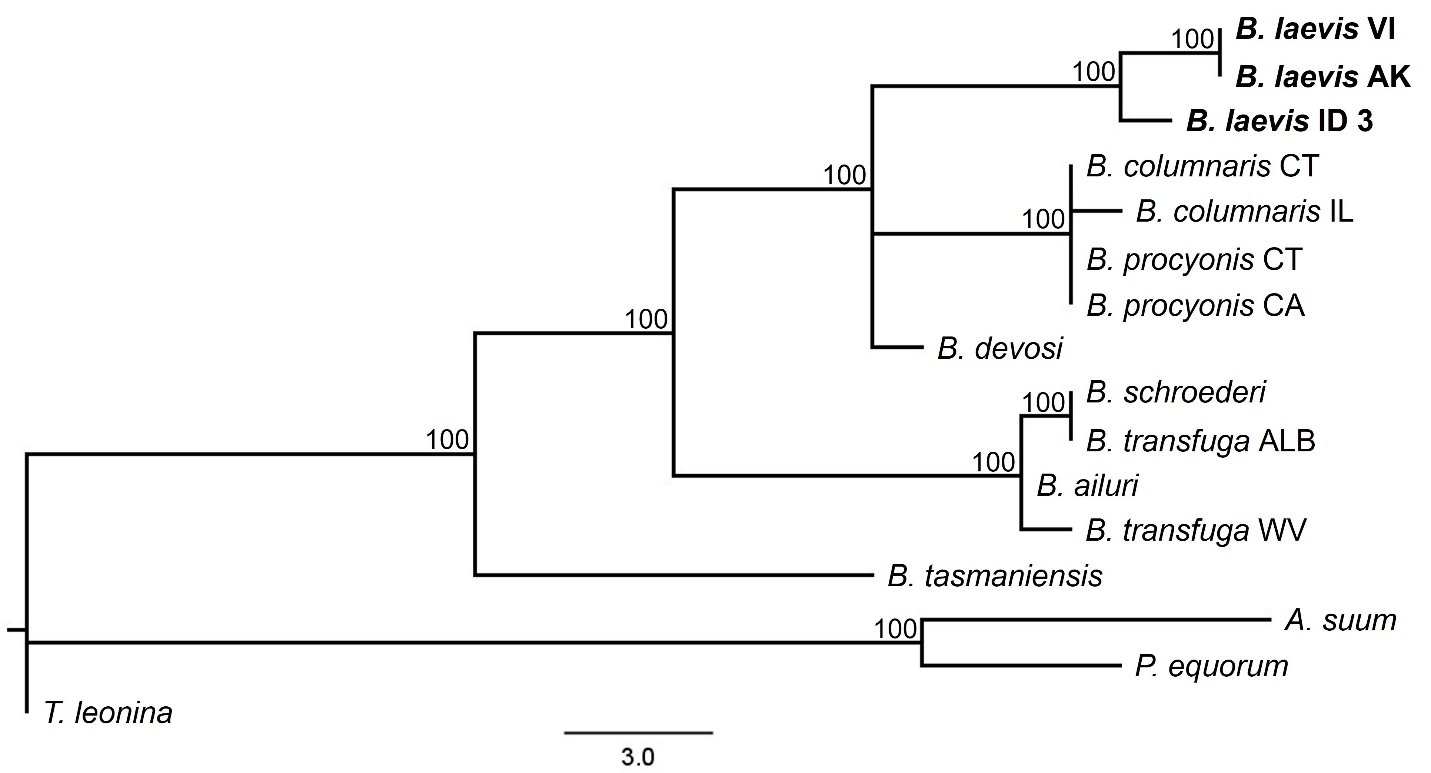
**

**Figure S10.** Phylogram based on strict consensus of maximum parsimony (MP) trees for *Baylisascaris* 28S. Branches are scaled to reflect character state substitutions. Bootstrap values above 0.90 are provided above each node.


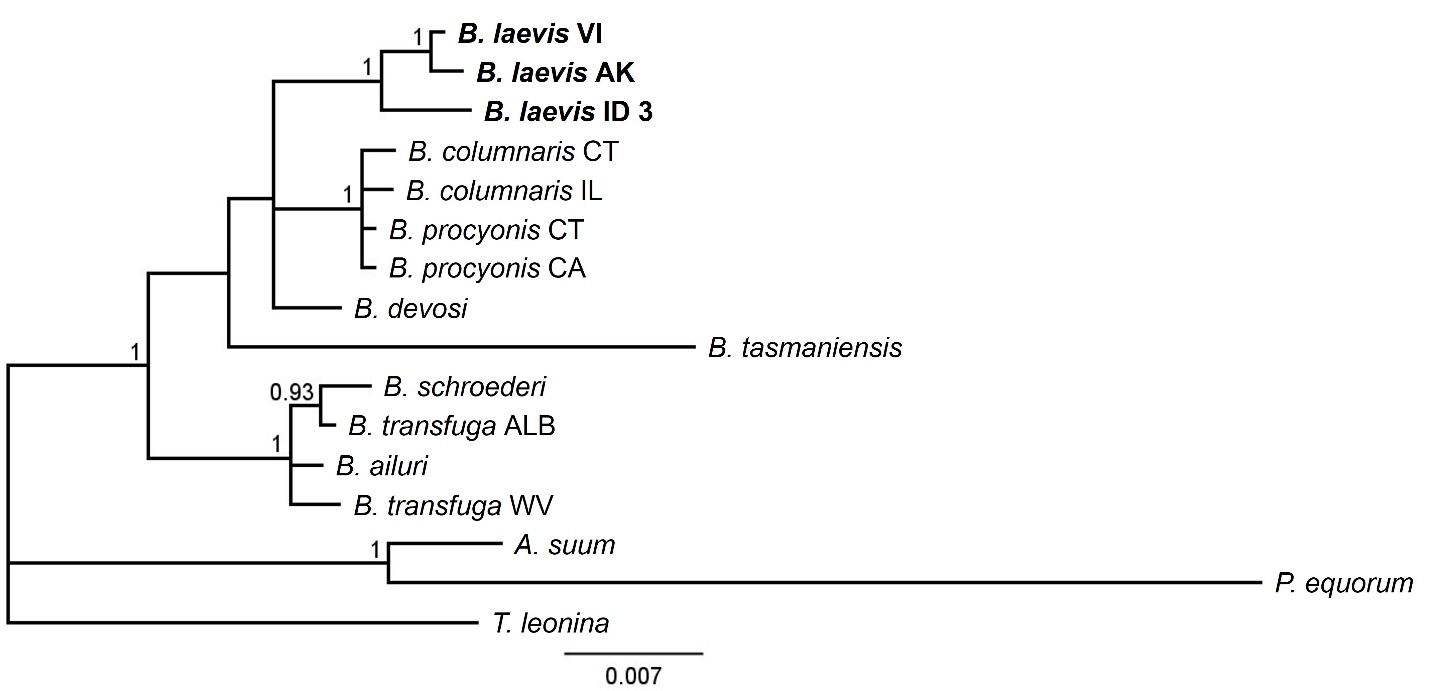


**Figure S11.** Bayesian consensus phylogram based on *Baylisascaris* and outgroup alignments of 28S. Branch labels represent Bayesian posterior probabilities. Branch lengths are scaled to expected number of substitutions per site.


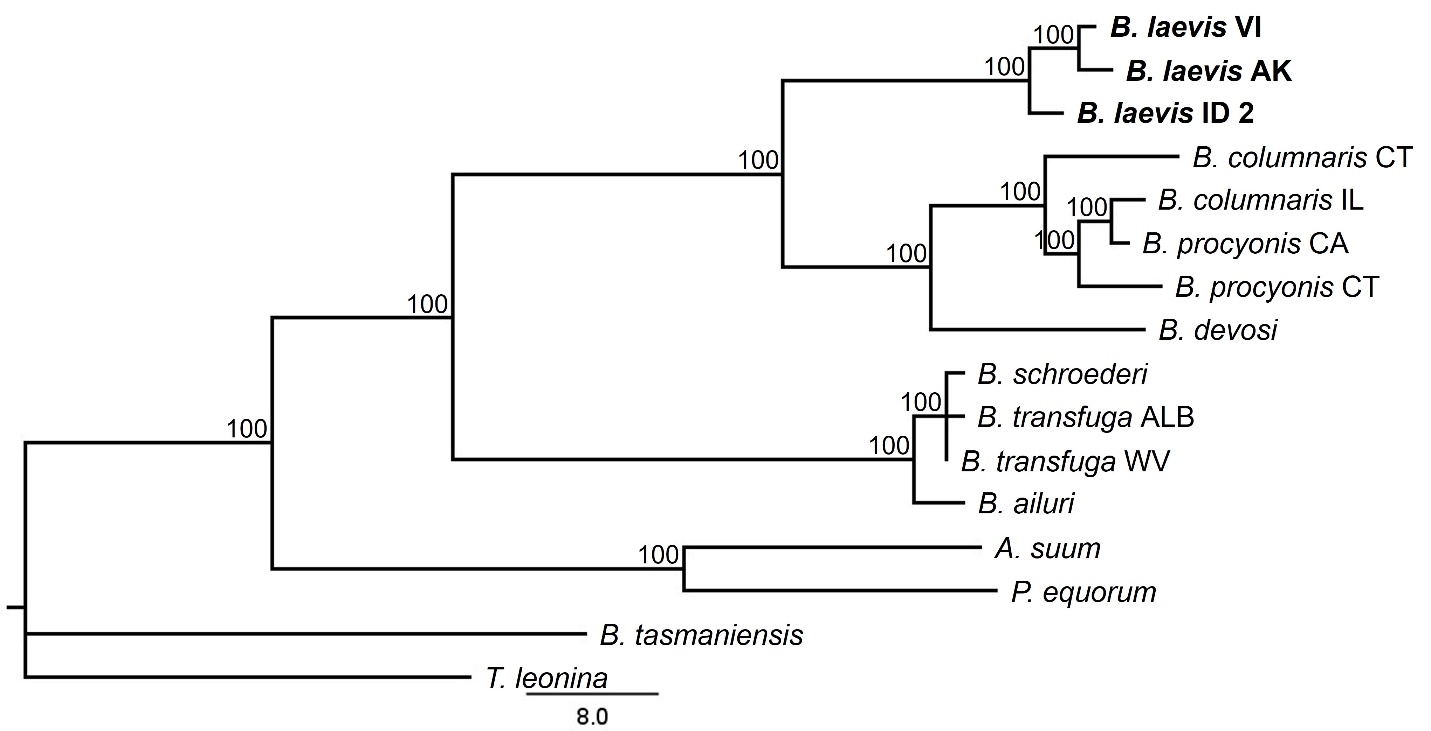


**Figure S12.** Phylogram based on strict consensus of maximum parsimony (MP) trees for *Baylisascaris ard1*. Branches are scaled to reflect character state substitutions. Bootstrap values above 0.90 are provided above each node.


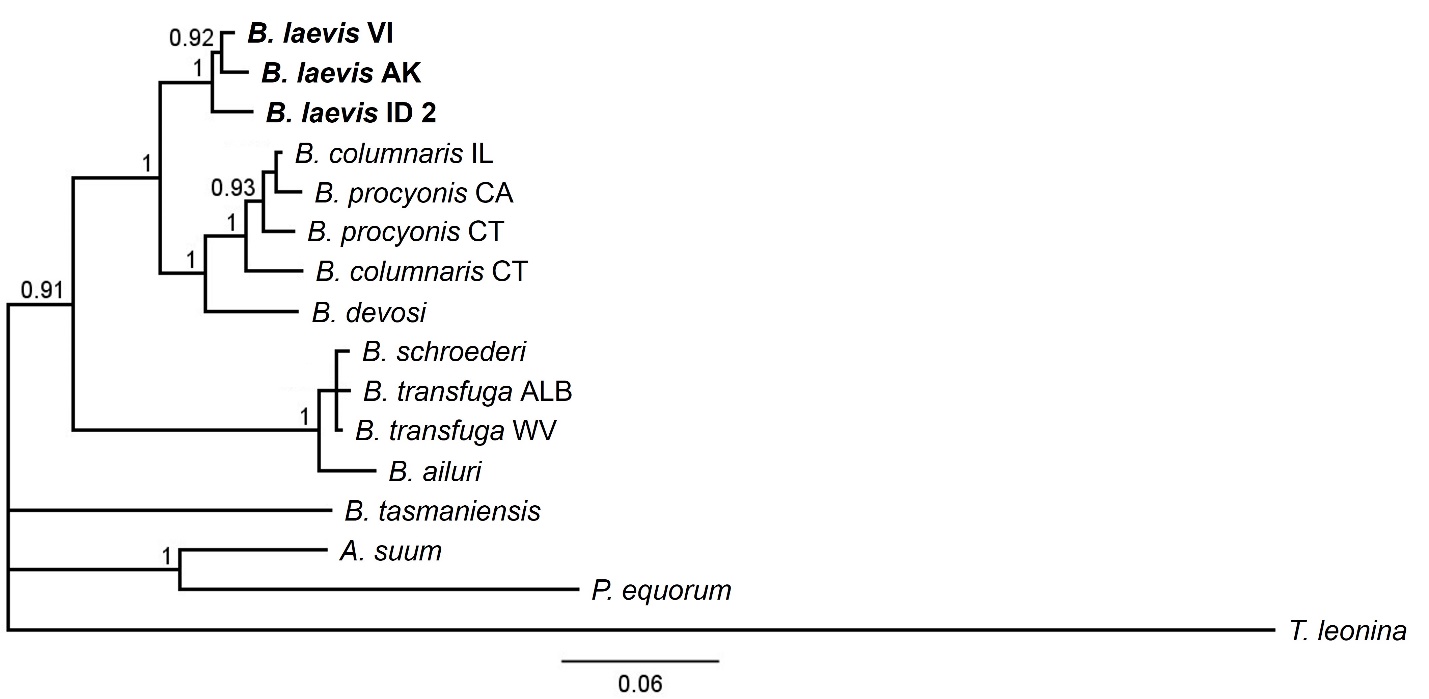


**Figure S13.** Bayesian consensus phylogram based on *Baylisascaris* and outgroup alignments of *ard1*. Branch labels represent Bayesian posterior probabilities. Branch lengths are scaled to expected number of substitutions per site.


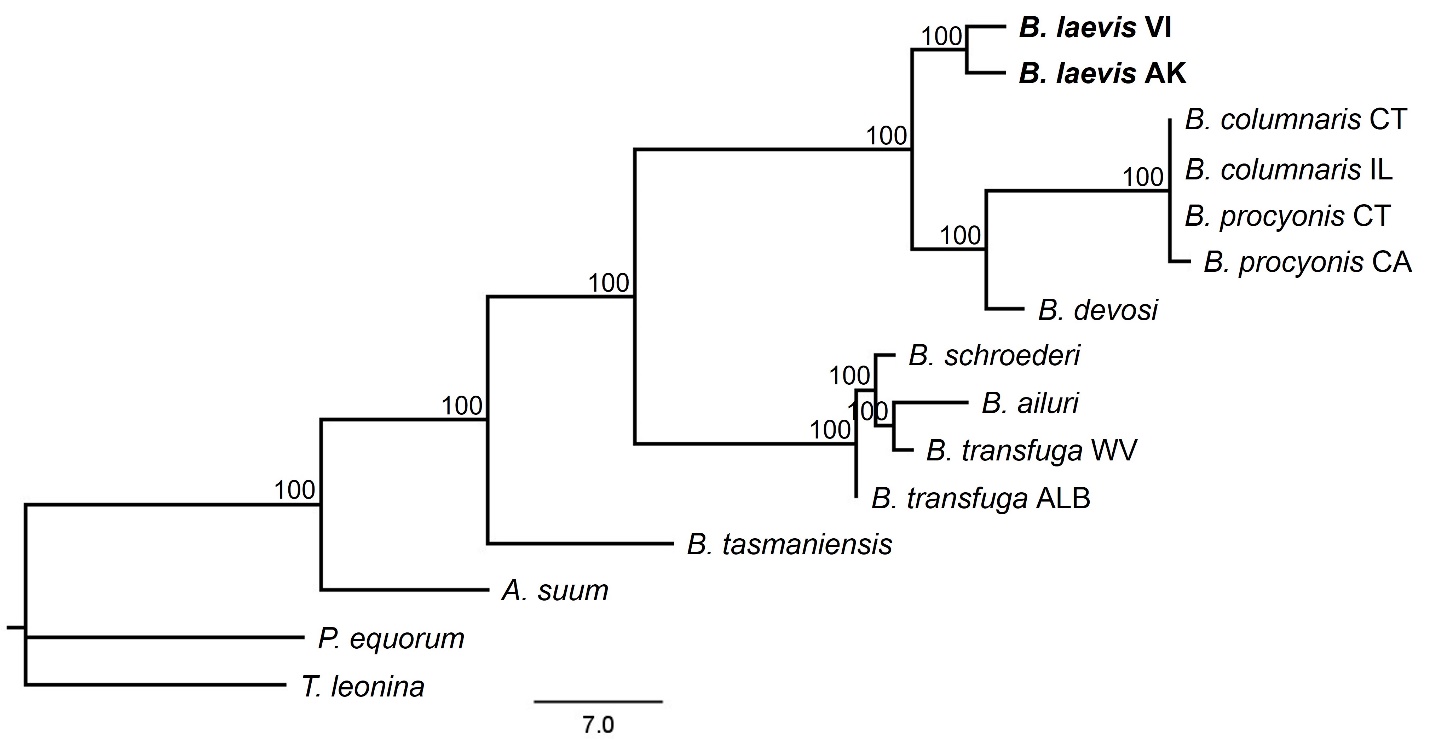


**Figure S14.** Phylogram based on strict consensus of maximum parsimony (MP) trees for *Baylisascaris* ITS. Branches are scaled to reflect character state substitutions. Bootstrap values above 0.90 are provided above each node.


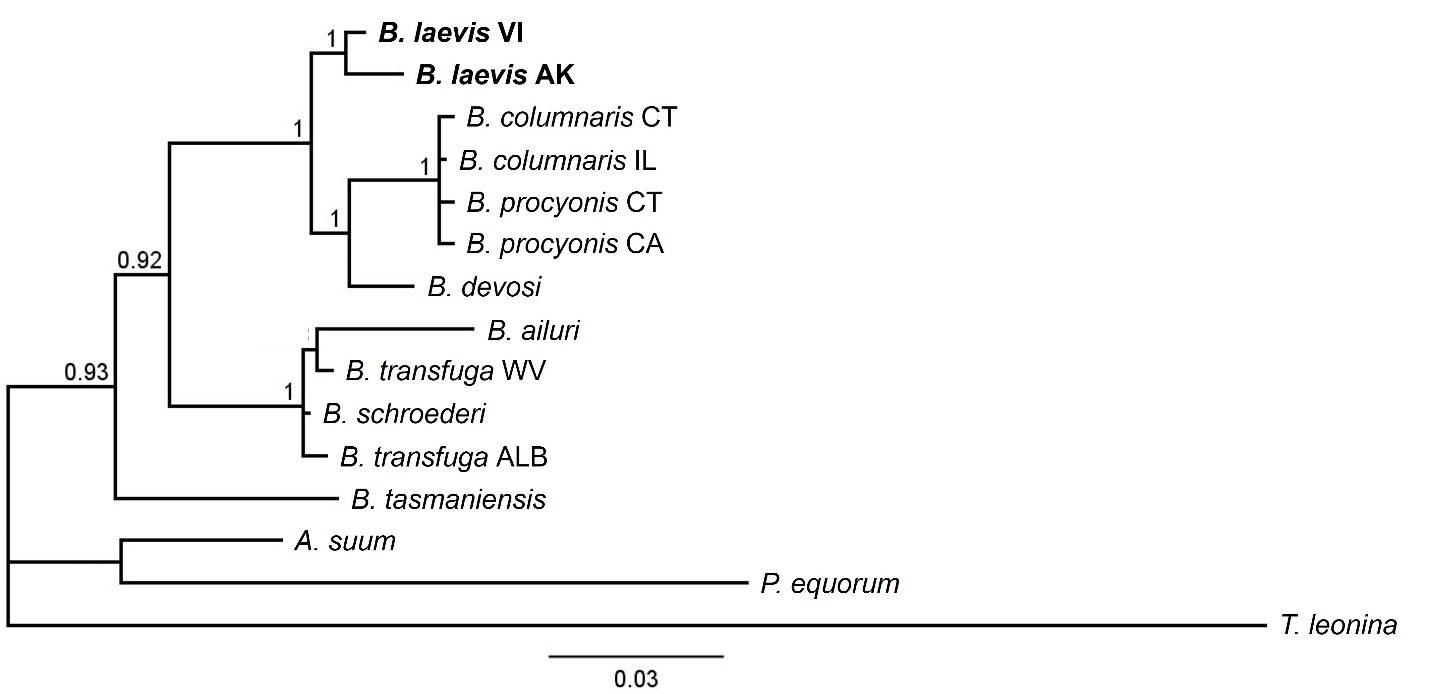


**Figure S15.** Bayesian consensus phylogram based on *Baylisascaris* and outgroup alignments of ITS. Branch labels represent Bayesian posterior probabilities. Branch lengths are scaled to expected number of substitutions per site.


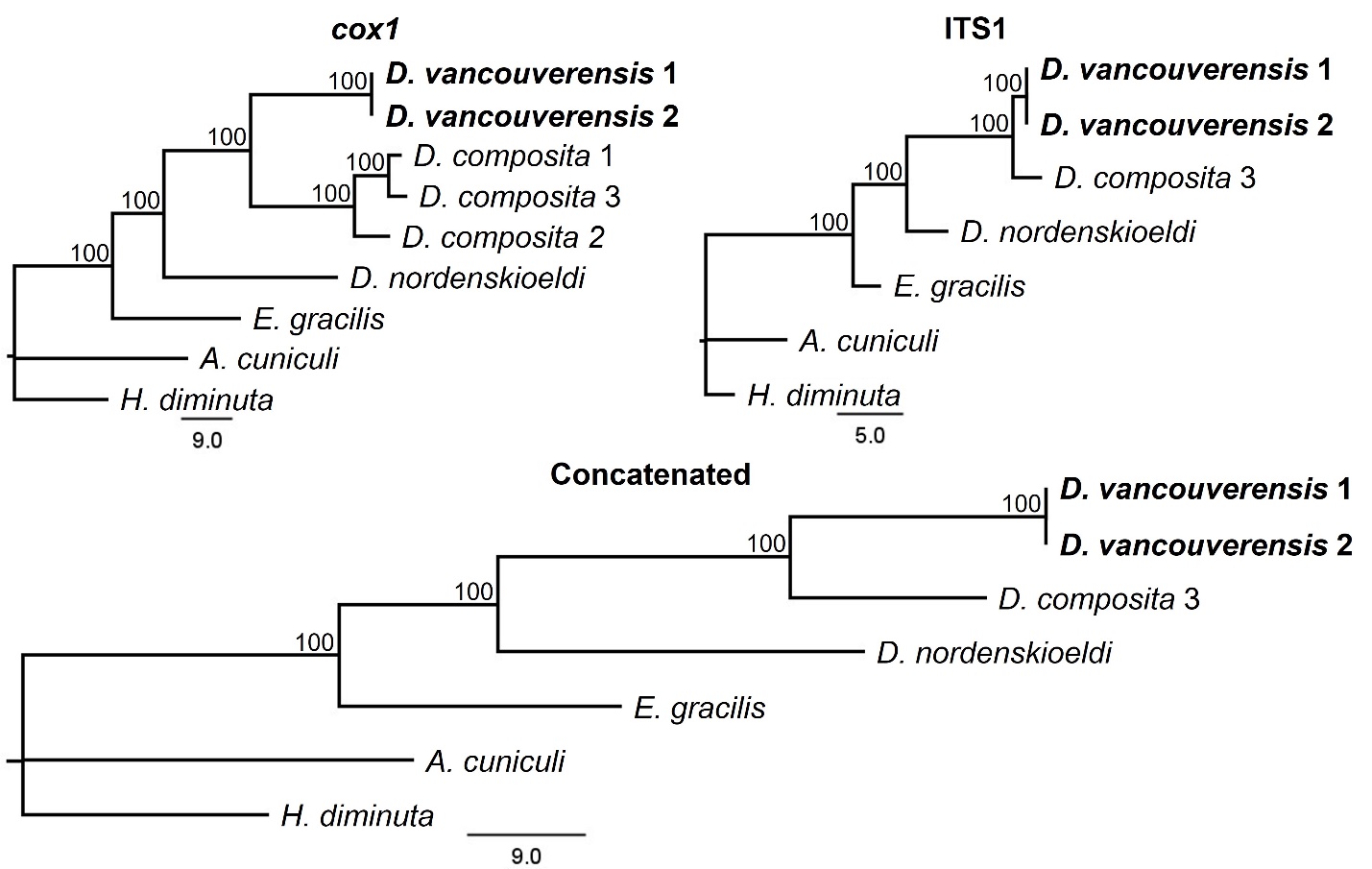


**Figure S16.** Phylogram based on strict consensus of maximum parsimony (MP) trees from *Diandrya* and outgroup alignments of concatenated loci *cox1* and ITS1. Branches are scaled to reflect character state substitutions. Bootstrap values above 0.90 are provided above each node.
